# Supplementary material for: Interplay of RNA m6A Modification-Related Geneset in Pan-Cancer
Source: Biomedicines. 2024 Sep 27;12(10):2211. doi: 10.3390/biomedicines12102211 (PMC11504890; doi:10.3390/biomedicines12102211)
Supplement: Supplementary file 1 [file biomedicines-12-02211-s001.zip › Supplementary Files/2024-9-3 2nd round revised biomedicines-3060735-supplementary.docx]

**Supplementary Table 1. Regulators of RNA m^6^A modification.**

| Gene | Full name | Kind | Reference |
| --- | --- | --- | --- |
| METTL3 | methyltransferase 3, N6-adenosine-methyltransferase complex catalytic subunit | writer | Ping et al., Cell Res. 2014 |
| METTL14 | methyltransferase 14, N6-adenosine-methyltransferase non-catalytic subunit | writer | Ping et al., Cell Res. 2014 |
| METTL16 | methyltransferase 16, RNA N6-adenosine | writer | Mendel et al., Mol Cell. 2018 |
| WTAP | WT1 associated protein | writer | Ping et al., Cell Res. 2014 |
| KIAA1429 (VIRMA) | vir like m6A methyltransferase associated | writer | Schwartz et al., *Cell Rep*. 2014 |
| ZC3H13 | zinc finger CCCH-type containing 13 | writer | Wen et al., *Mol Cell*. 2018 |
| CBLL1 (HAKAI) | Cbl proto-oncogene like 1 | writer | Růžička et al., *New Phytol*. 2017 |
| RBM15 | RNA binding motif protein 15 | writer | Patil et al., *Nature*. 2016 |
| RBM15B | RNA binding motif protein 15B | writer | Patil et al., *Nature*. 2016 |
| METTL5 | methyltransferase 5, N6-adenosine | writer | van Tran et al., *Nucleic Acids Res*. 2019 |
| TRMT112 | tRNA methyltransferase activator subunit 11-2 | writer | van Tran et al., *Nucleic Acids Res*. 2019 |
| ZCCHC4 | zinc finger CCHC-type containing 4 | writer | van Tran et al., *Nucleic Acids Res*. 2019 |
| FTO | FTO alpha-ketoglutarate dependent dioxygenase | eraser | Jia et al., *Nat Chem Biol*. 2011 |
| ALKBH5 | alkB homolog 5, RNA demethylase | eraser | Zheng et al., *Mol Cell*. 2013 |
| YTHDC1 | YTH N6-methyladenosine RNA binding protein C1 | reader | Xiao et al., *Mol Cell*. 2016 |
| YTHDC2 | YTH N6-methyladenosine RNA binding protein C2 | reader | Hsu et al., *Cell Res*. 2017 |
| YTHDF1 | YTH N6-methyladenosine RNA binding protein F1 | reader | Wang et al., *Cell*. 2015 |
| YTHDF2 | YTH N6-methyladenosine RNA binding protein F2 | reader | Wang et al., *Nature*. 2014 |
| YTHDF3 | YTH N6-methyladenosine RNA binding protein F3 | reader | Li et al., *Cell Res*. 2017 |
| HNRNPC | heterogeneous nuclear ribonucleoprotein C | reader | Liu et al., *Nature*. 2015 |
| RBMX (HNRNPG) | RNA binding motif protein X-linked | reader | Liu et al., *Nucleic Acids Res*. 2017 |
| FMR1 | fragile X messenger ribonucleoprotein 1 | reader | Edupuganti et al., *Nat Struct Mol Biol*. 2017 |
| HNRNPA2B1 | heterogeneous nuclear ribonucleoprotein A2/B1 | reader | Alarcón et al., *Cell*. 2015 |
| IGF2BP1 | insulin like growth factor 2 mRNA binding protein 1 | reader | Huang et al., *Nat Cell Biol*. 2018 |
| IGF2BP2 | insulin like growth factor 2 mRNA binding protein 2 | reader | Huang et al., *Nat Cell Biol*. 2018 |
| IGF2BP3 | insulin like growth factor 2 mRNA binding protein 3 | reader | Huang et al., *Nat Cell Biol*. 2018 |
| PRRC2A | proline rich coiled-coil 2A | reader | Wu et al., *Cell Res*. 2019 |
| RBM33 | RNA binding motif protein 33 | reader | Yu et al., *Mol Cell*. 2023 |
| RBFOX2 | RNA binding fox-1 homolog 2 | reader | Dou et al., *Nat Cell Biol*. 2023 |
| LRPPRC | leucine rich pentatricopeptide repeat containing | reader | Arguello et al., *J Am Chem Soc*. 2017 |
| FXR1 | FMR1 autosomal homolog 1 | reader | Deng et al., *Nat Genet*. 2022 |

**Supplementary Table 2. 28 types of cancers in TCGA database.**

| Abbreviation | Full name | Number of patients in TCGA cohort |
| --- | --- | --- |
| ACC | Adrenocortical carcinoma | 79 |
| BLCA | Bladder Urothelial Carcinoma | 408 |
| BRCA | Breast invasive carcinoma | 1098 |
| CESC | Cervical squamous cell carcinoma and endocervical adeno carcinoma | 306 |
| CHOL | Cholangio carcinoma | 36 |
| COAD | Colon adenocarcinoma | 458 |
| ESCA | Esophageal carcinoma | 162 |
| GBM | Glioblastoma multiforme | 167 |
| HNSC | Head and Neck squamous cell carcinoma | 502 |
| KICH | Kidney Chromophobe | 65 |
| KIRC | Kidney renal clear cell carcinoma | 531 |
| KIRP | Kidney renal papillary cell carcinoma | 289 |
| LAML | Acute Myeloid Leukemia | 151 |
| LGG | Brain Lower Grade Glioma | 525 |
| LIHC | Liver hepatocellular carcinoma | 373 |
| LUAD | Lung adenocarcinoma | 515 |
| LUSC | Lung squamous cell carcinoma | 501 |
| OV | Ovarian serous cystadenocarcinoma | 379 |
| PAAD | Pancreatic adenocarcinoma | 178 |
| PCPG | Pheochromocytoma and Paraganglioma | 183 |
| PRAD | Prostate adenocarcinoma | 496 |
| READ | Rectum adenocarcinoma | 167 |
| SKCM | Skin Cutaneous Melanoma | 471 |
| STAD | Stomach adenocarcinoma | 375 |
| TGCT | Testicular Germ Cell Tumors | 156 |
| THCA | Thyroid carcinoma | 510 |
| UCEC | Uterine Corpus Endometrial Carcinoma | 544 |
| UCS | Uterine Carcinosarcoma | 56 |

**Supplementary Table 3. 45 types of normal tissue in TCGA and GTEx database.**

| Normal tissues | | |
| --- | --- | --- |
| Vagina | Prostate | Esophagus |
| Urinary bladder | Placenta | Epididymis |
| Tonsil | Parathyroid gland | Endometrium |
| Thyroid gland | Pancreas | Duodenum |
| Testis | Ovary | Colon |
| Stomach | Oral mucosa | Cervix |
| Spleen | Nasopharynx | Cerebral cortex |
| Soft tissue | Lymph node | Cerebellum |
| Smooth muscle | Lung | Bronchus |
| Small intestine | Liver | Breast |
| Skin | Kidney | Bone marrow |
| Skeletal muscle | Hippocampal formation | Basal ganglia |
| Seminal vesicle | Heart muscle | Appendix |
| Salivary gland | Gallbladder | Adrenal gland |
| Rectum | Fallopian tube | Adipose tissue |


**Supplementary Table 4. Patients’ number distribution of clinicopathological characteristics in each cancer cohort**

|  | ACC | BLCA | BRCA | CESC | COAD | CHOL | ESCA | GBM | HNSC | KIRP | KIRC | KICH | LGG | LUAD | LUSC | LIHC | LAML | OV | PRAD | PAAD | PCPG | READ | STAD | SKCM | THCA | TGCT | UCEC | UCS |
| --- | --- | --- | --- | --- | --- | --- | --- | --- | --- | --- | --- | --- | --- | --- | --- | --- | --- | --- | --- | --- | --- | --- | --- | --- | --- | --- | --- | --- |
| Young | 57 | 87 | 580 | 239 | 92 | 12 | 82 | 71 | 232 | 120 | 243 | 47 | 439 | 137 | 90 | 168 | 90 | 220 | 201 | 55 | 136 | 37 | 122 | 37 | 384 | 130 | 48 | 6 |
| Old | 20 | 320 | 510 | 65 | 194 | 24 | 99 | 81 | 285 | 165 | 287 | 19 | 69 | 357 | 399 | 200 | 83 | 199 | 294 | 123 | 41 | 54 | 287 | 65 | 120 | 2 | 129 | 51 |
| Male | 31 | 301 | 12 | 0 | 156 | 16 | 155 | 98 | 382 | 213 | 344 | 39 | 283 | 237 | 369 | 249 | 93 | 0 | 0 | 98 | 77 | 49 | 268 | 60 | 136 | 0 | 0 | 0 |
| Female | 46 | 106 | 1079 | 0 | 130 | 20 | 26 | 54 | 136 | 75 | 186 | 27 | 225 | 276 | 129 | 120 | 80 | 0 | 0 | 80 | 100 | 42 | 146 | 42 | 368 | 0 | 0 | 0 |
| G1 | 0 | 0 | 0 | 18 | 0 | 0 | 18 | 0 | 61 | 0 | 14 | 0 | 0 | 0 | 0 | 55 | 0 | 0 | 0 | 31 | 0 | 0 | 12 | 0 | 0 | 0 | 14 | 0 |
| G2 | 0 | 0 | 0 | 135 | 0 | 15 | 74 | 0 | 304 | 0 | 228 | 0 | 247 | 0 | 0 | 177 | 0 | 47 | 0 | 95 | 0 | 0 | 148 | 0 | 0 | 0 | 21 | 0 |
| G3 | 0 | 0 | 0 | 118 | 0 | 18 | 49 | 0 | 124 | 0 | 206 | 0 | 260 | 0 | 0 | 121 | 0 | 360 | 0 | 48 | 0 | 0 | 245 | 0 | 0 | 0 | 141 | 0 |
| G4 | 0 | 0 | 0 | 0 | 0 | 0 | 0 | 0 | 7 | 0 | 74 | 0 | 0 | 0 | 0 | 11 | 0 | 0 | 0 | 0 | 0 | 0 | 0 | 0 | 0 | 0 | 0 | 0 |
| Stage I | 9 | 0 | 182 | 162 | 44 | 19 | 18 | 0 | 27 | 177 | 266 | 21 | 0 | 274 | 242 | 169 | 0 | 0 | 0 | 21 | 0 | 12 | 58 | 0 | 283 | 104 | 98 | 22 |
| Stage II | 36 | 130 | 617 | 69 | 110 | 9 | 80 | 0 | 82 | 25 | 57 | 25 | 0 | 122 | 161 | 86 | 0 | 24 | 0 | 147 | 0 | 24 | 121 | 66 | 52 | 13 | 24 | 5 |
| Stage III | 15 | 140 | 248 | 45 | 82 | 0 | 61 | 0 | 93 | 52 | 123 | 14 | 0 | 83 | 84 | 85 | 0 | 328 | 0 | 3 | 0 | 33 | 169 | 26 | 112 | 14 | 48 | 20 |
| Stage IV | 15 | 133 | 20 | 21 | 40 | 7 | 16 | 0 | 316 | 16 | 81 | 6 | 0 | 26 | 7 | 5 | 0 | 63 | 0 | 4 | 0 | 13 | 41 | 3 | 55 | 0 | 10 | 10 |
| T1 | 9 | 3 | 280 | 140 | 6 | 19 | 31 | 0 | 51 | 191 | 272 | 21 | 0 | 169 | 112 | 179 | 0 | 0 | 0 | 7 | 0 | 4 | 21 | 0 | 142 | 76 | 0 | 0 |
| T2 | 41 | 150 | 630 | 71 | 44 | 12 | 43 | 0 | 157 | 33 | 69 | 25 | 0 | 276 | 292 | 94 | 0 | 0 | 188 | 24 | 0 | 13 | 89 | 5 | 166 | 50 | 0 | 0 |
| T3 | 8 | 195 | 138 | 20 | 196 | 5 | 97 | 0 | 120 | 60 | 179 | 18 | 0 | 47 | 71 | 80 | 0 | 0 | 292 | 142 | 0 | 63 | 180 | 10 | 171 | 6 | 0 | 0 |
| T4 | 17 | 58 | 40 | 10 | 40 | 0 | 6 | 0 | 189 | 0 | 10 | 0 | 0 | 18 | 23 | 13 | 0 | 0 | 10 | 3 | 0 | 10 | 115 | 84 | 23 | 0 | 0 | 0 |
| N0 | 66 | 237 | 514 | 133 | 166 | 26 | 76 | 0 | 220 | 144 | 240 | 40 | 0 | 329 | 317 | 250 | 0 | 0 | 343 | 49 | 0 | 38 | 124 | 58 | 229 | 79 | 0 | 0 |
| N1 | 9 | 46 | 361 | 60 | 71 | 5 | 78 | 0 | 78 | 25 | 15 | 3 | 0 | 96 | 130 | 4 | 0 | 0 | 79 | 124 | 0 | 29 | 111 | 8 | 225 | 22 | 0 | 0 |
| N2 | 0 | 74 | 119 | 0 | 49 | 0 | 13 | 0 | 205 | 4 | 0 | 0 | 0 | 74 | 40 | 0 | 0 | 0 | 0 | 0 | 0 | 21 | 78 | 10 | 0 | 3 | 0 | 0 |
| N3 | 0 | 8 | 77 | 0 | 0 | 0 | 7 | 0 | 11 | 0 | 0 | 0 | 0 | 0 | 5 | 0 | 0 | 0 | 0 | 0 | 0 | 0 | 82 | 10 | 0 | 4 | 0 | 0 |
| M0 | 60 | 196 | 907 | 116 | 193 | 28 | 141 | 0 | 502 | 206 | 441 | 0 | 0 | 344 | 408 | 264 | 0 | 0 | 453 | 80 | 0 | 63 | 366 | 97 | 281 | 126 | 0 | 0 |
| M1 | 15 | 11 | 22 | 10 | 40 | 5 | 16 | 0 | 5 | 12 | 79 | 0 | 0 | 25 | 7 | 4 | 0 | 0 | 3 | 4 | 0 | 12 | 27 | 3 | 9 | 6 | 0 | 0 |

**Supplementary Table 5. Prognosis related genes in each tumor type based on LASSO cox regression**

| **Cancer type** | **Prognosis related genes based on LASSO regression** |
| --- | --- |
| ACC | *YTHDF1, EIF3A, HNRNPA2B1, FTO, LRPPRC, METTL14, ELAVL1, ZC3H13, YTHDF3* |
| BLCA | *FTO, ZC3H13, FXR1, FMR1, LRPPRC, FXR2, G3BP1, EIF3A, ALKBH5, HNRNPA2B1, YTHDF3, CBLL1, YTHDC1* |
| BRCA | *METTL16, IGF2BP1, ABCF1, YTHDF3, EIF4G2, RBM15B, EIF3A, HNRNPC* |
| CESC | *ZC3H13, G3BP1, FMR1* |
| COAD | *FXR1, IGF2BP3, YTHDC2* |
| ESCA | *FMR1, ALKBH5, IGF2BP2, HNRNPC* |
| KICH | *FTO, FXR1, G3BP1, HNRNPC, EIF3A, ABCF1, IGF2BP2, RBM15B, METTL3, RBM15, YTHDC1, ELAVL1, METTL16, YTHDC2* |
| KIRC | *ALKBH5, VIRMA, YTHDF2, IGF2BP3, IGF2BP2, ELAVL1 , FXR1, HNRNPA2B1, IGF2BP1, LRPPRC, EIF3A, YTHDC1, METTL14* |
| KIRP | *ALKBH5, FXR1* |
| LAML | *ELAVL1, RBM15, ZC3H13, YTHDF3, FXR1, YTHDC2, FMR1, IGF2BP2, EIF4G2, IGF2BP3, YTHDF1, ALKBH5, RBM15B, FXR2, HNRNPC, ABCF1, EIF3A, RBMX, YTHDC1, HNRNPA2B1* |
| LGG | *METTL16, FXR1, RBM15, G3BP1, VIRMA, HNRNPC, HNRNPA2B1, IGF2BP1, IGF2BP3, YTHDF1, FMR1, METTL3, YTHDF2, LRPPRC, YTHDC2* |
| LIHC | *YTHDF2, LRPPRC, YTHDF1, FTO, VIRMA, ZC3H13, CBLL1, WTAP, EIF4G2, HNRNPA2B1, ALKBH5, IGF2BP1, FXR1* |
| LUAD | *METTL16, YTHDF2, HNRNPA2B1* |
| PAAD | *FXR1, VIRMA, METTL3, METTL16, HNRNPC, YTHDC2, IGF2BP3, YTHDF1, RBMX, ABCF1, METTL14* |
| READ | *FTO, RBM15* |
| UCS | *IGF2BP1, YTHDF2* |

**Supplementary Table 6. Prognosis related genes in each tumor type based on CNV data with three machine learning algorithms**

| **Cancer type** | **Prognosis related genes based on CNV data** |
| --- | --- |
| BLCA | *IGF2BP3, FTO, YTHDF3* |
| CHOL | *YTHDC1, FXR1, ZC3H13, FTO, METTL14, WTAP, RBM15, VIRMA* |
| GBM | *IGF2BP2, RBFOX2, FTO* |
| KIRP | *RBFOX2, FTO, RBM15, TRMT112, YTHDF2, RBM15B* |
| LGG | *FMR1, CBLL1, FXR1, FTO, METTL3* |
| LUAD | *YTHDC2, FXR1, WTAP, RBM15, ZCCHC4* |
| OV | *YTHDC2, ALKBH5, FMR1, CBLL1, FXR1, HNRNPA2B1, ZC3H13, IGF2BP3, WTAP, RBM15, VIRMA, ZCCHC4, YTHDF2* |
| PCPG | *FTO, YTHDF2* |

**Supplementary Figure S1. Differential expression of IGF2BP2 (A) and IGF2BP3 (B) in various normal and cancer tissues.**


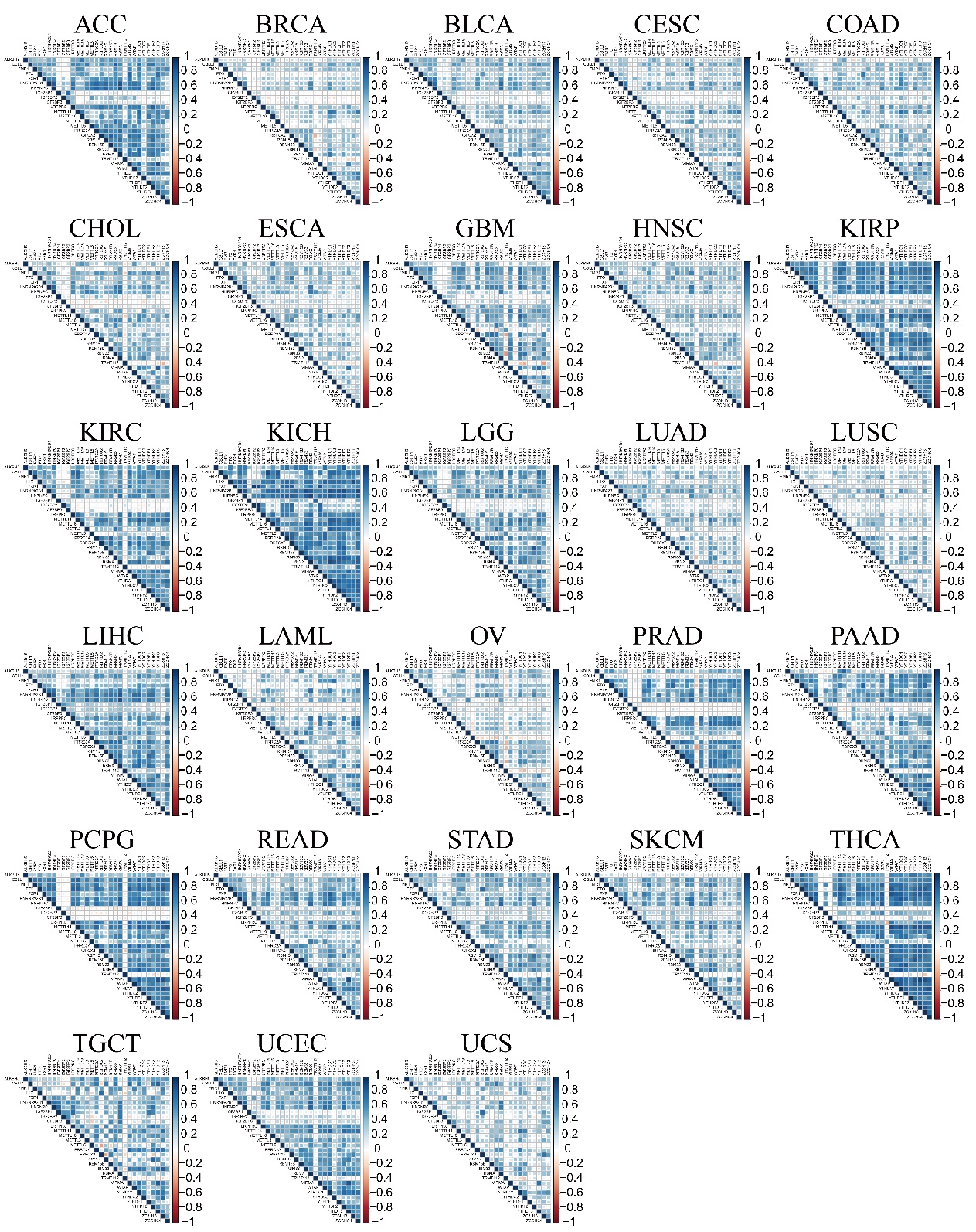


**Supplementary Figure S2. Correlation between m^6^A modification genes in 28 cancer types.**


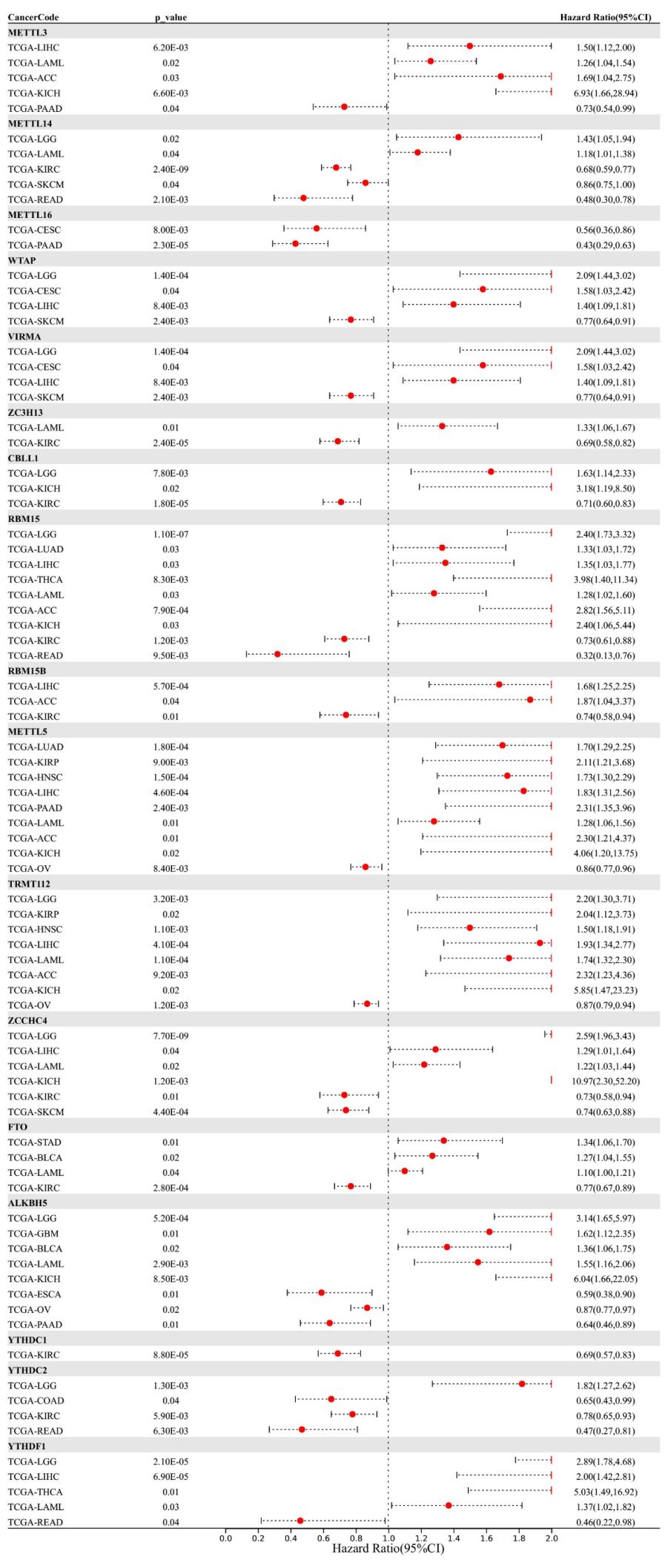


**Supplementary Figure S3. Survival analysis of m^6^A modification genes in 28 tumor types.**


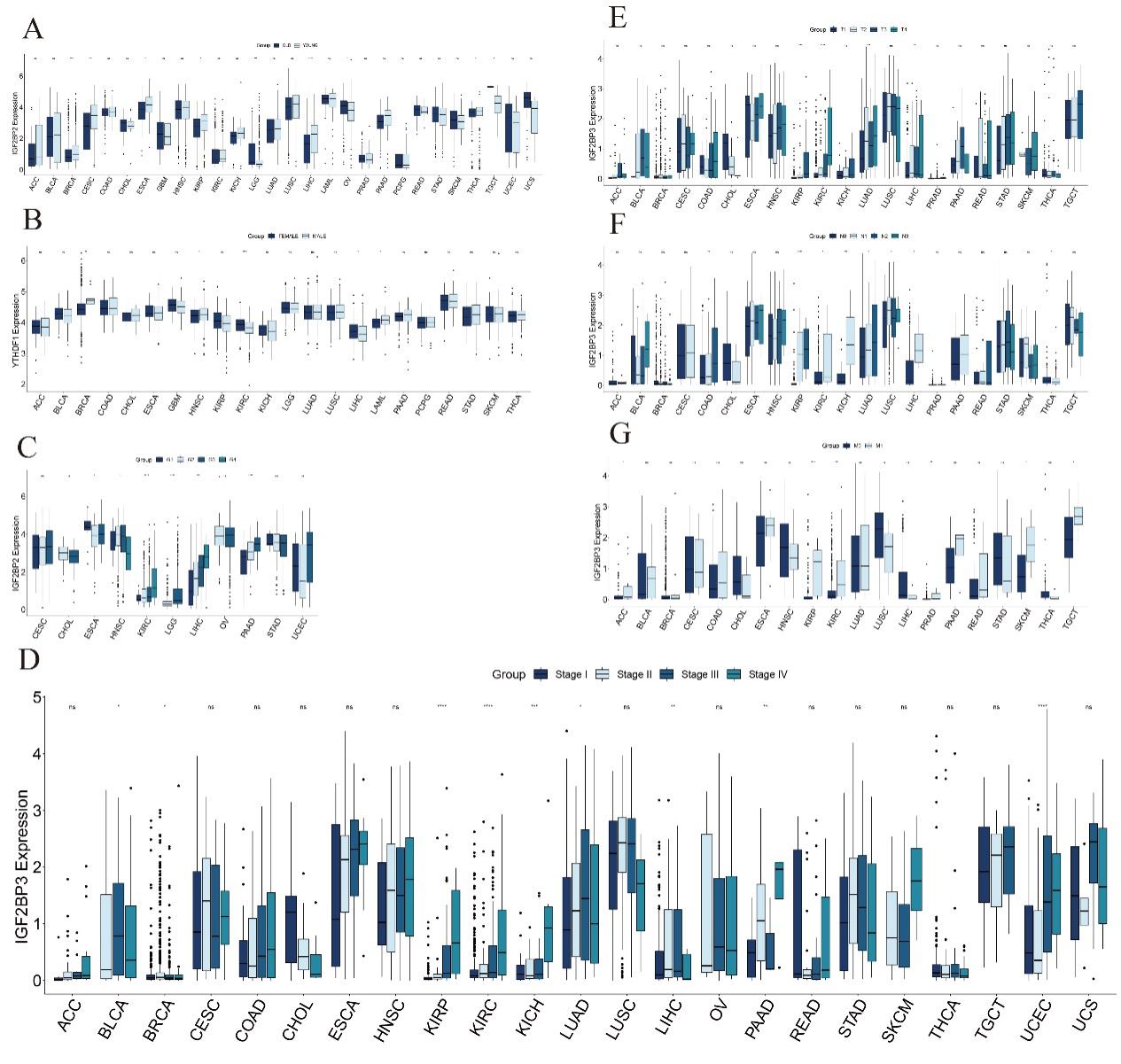


**Supplementary Figure S4. Clinicopathological characteristics of representative m^6^A modification genes in each cancer.** Differential expression of *IGF2BP2* between age (A) and grade (C) groups, (B) *YTHDF1* between gender groups, *IGF2BP3* between stage (D), T (E), N (F), and M (G) groups. (*p < 0.05, **p < 0.01, ***p < 0.001, ****p < 0.0001).


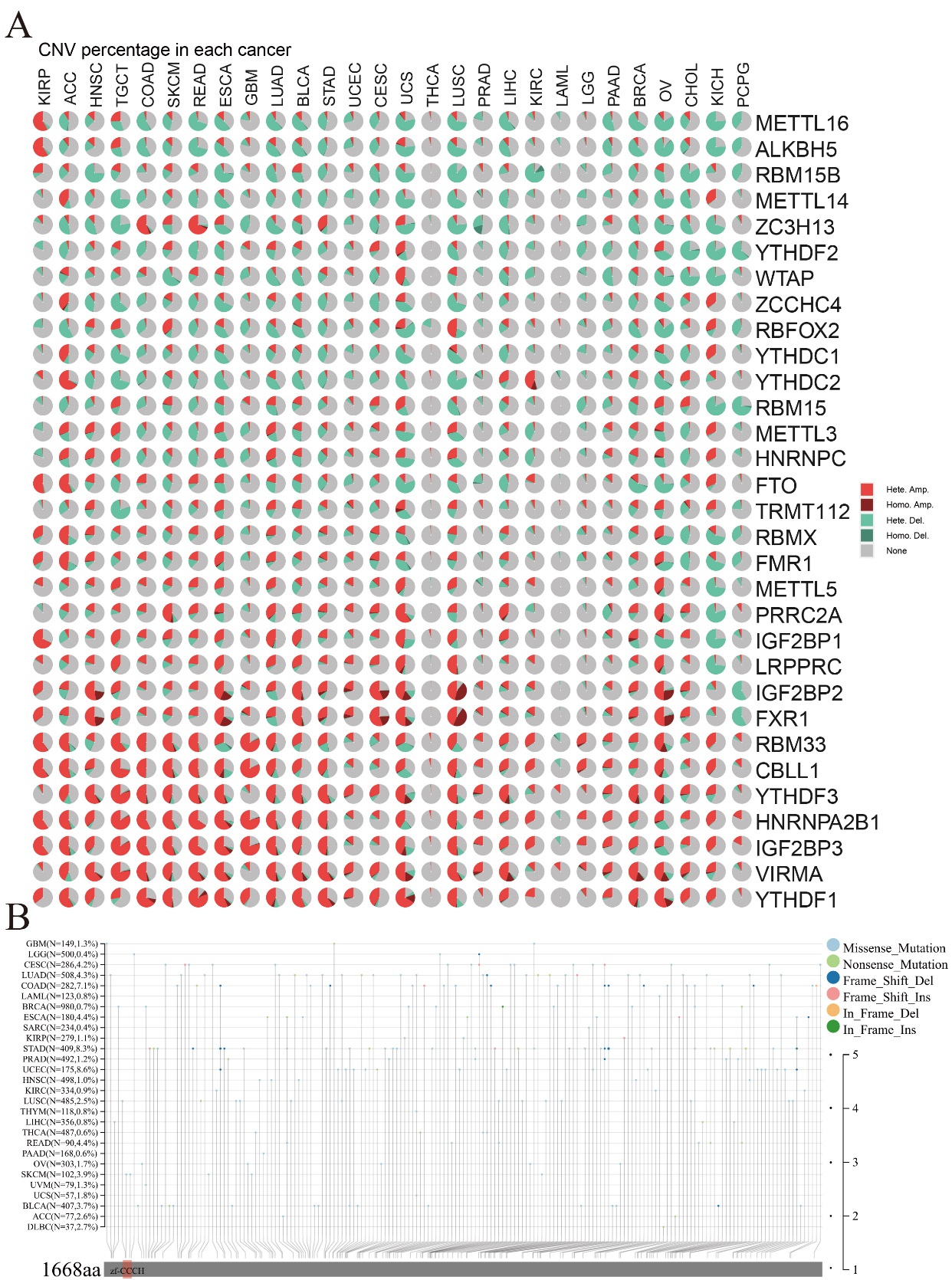


**Supplementary Figure S5. Genetic and epigenetic alterations of m^6^A modification genes in each cancer.** (A) CNV percentage in the regulator genes in each cancer. (B) Percentage of mutation of ZC3H13 in different cancer types.


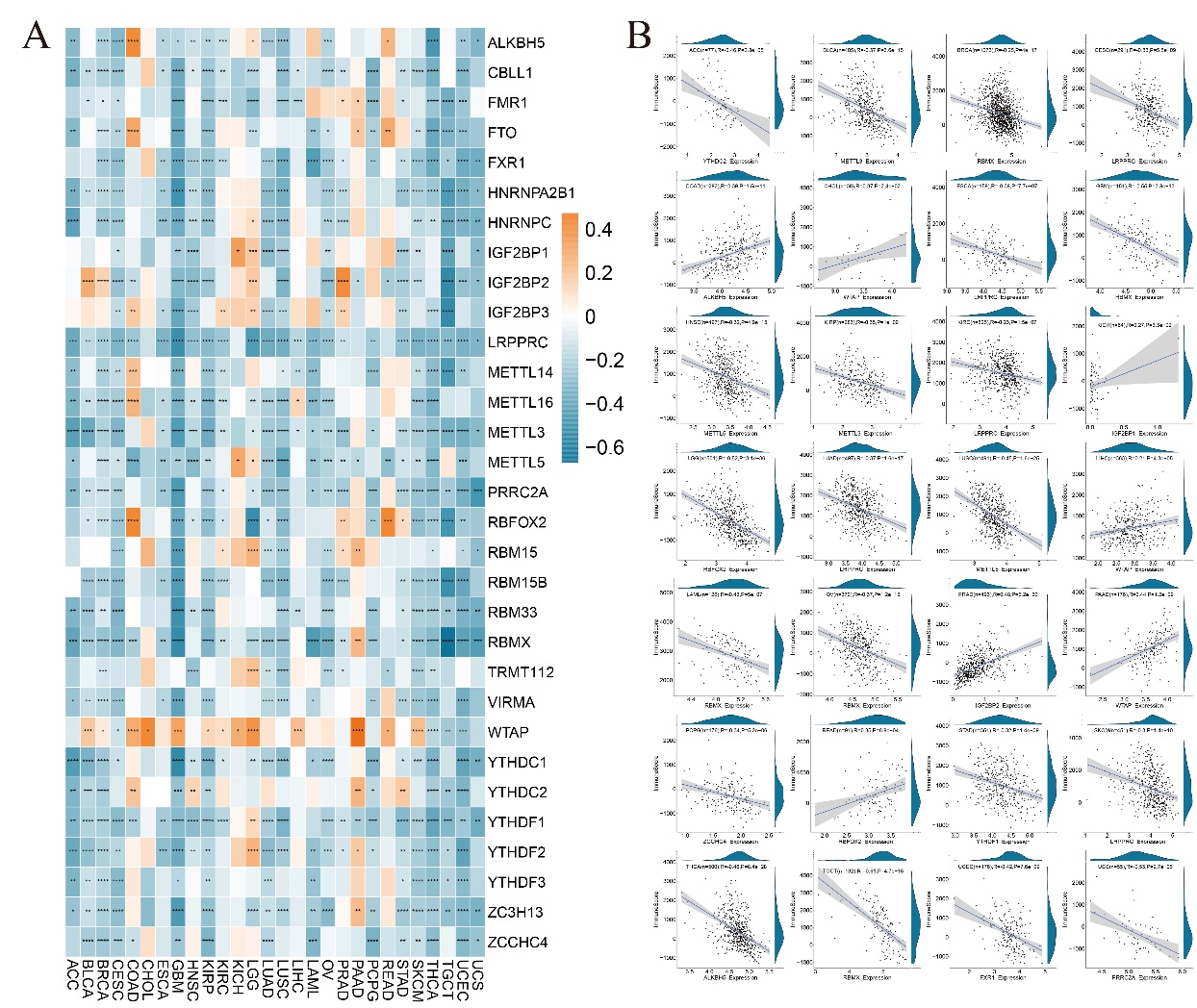


**Supplementary Figure S6. The relationship between m^6^A modification genes and** **ImmuneScore in** **multiple cancers.** (A) Heatmap plot showing the correlations between genes and ImmuneScore in each cancer. (B) Scatter plots showing the correlation coefficient and *p-*values between a few representative genes and ImmuneScore in selected cancers. (**p* < 0.05, ***p* < 0.01, ****p* < 0.001, *****p* < 0.0001).


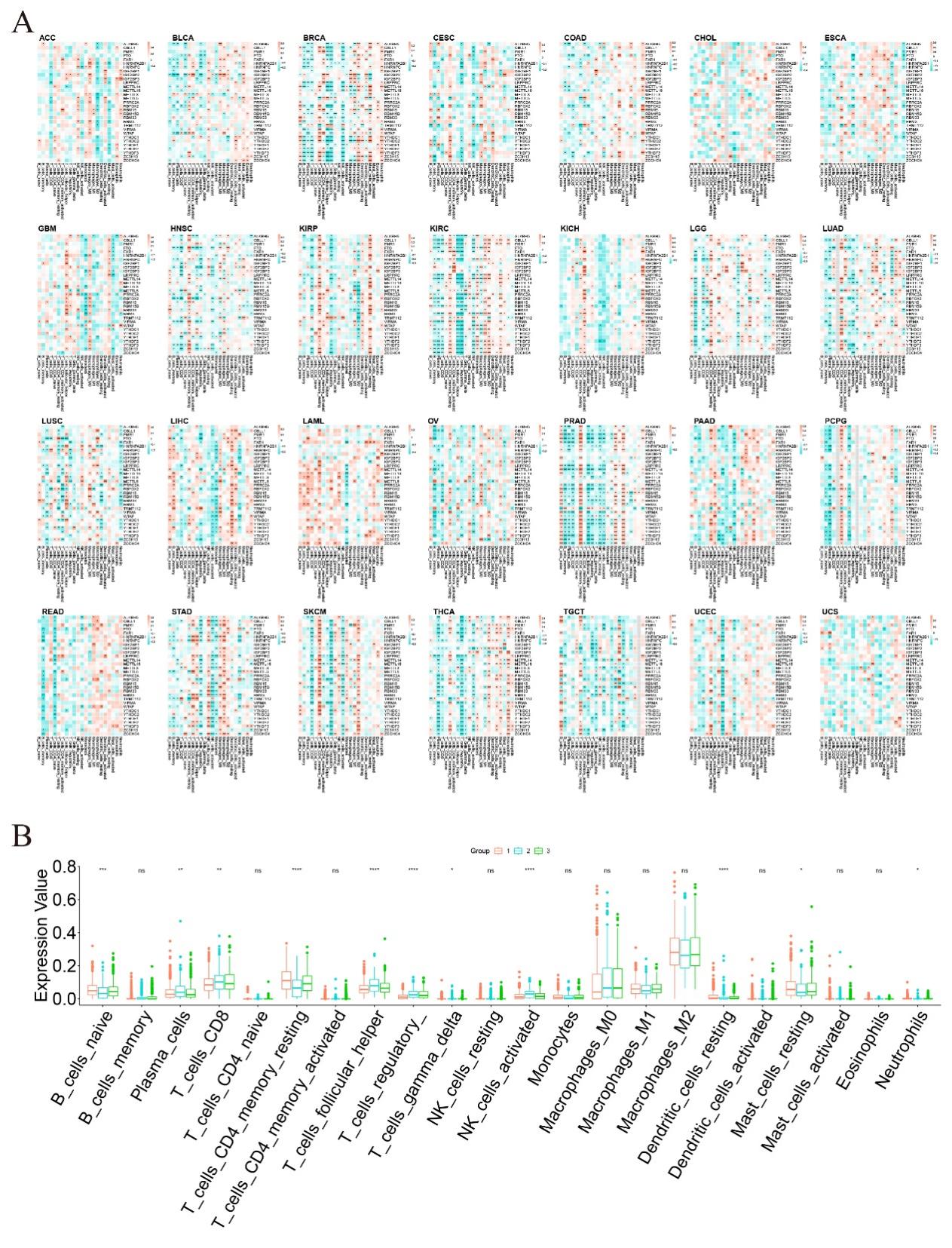


**Supplementary Figure S7. Association of immune cell infiltration with RNA m^6^A regulators in TCGA pan-cancer cohort.** (A) Correlation between m^6^A modification genes and immune cells. (B) Differential immune cell infiltration between clusters in BRCA. (**p* < 0.05, ***p* < 0.01, ****p* < 0.001, *****p* < 0.0001).


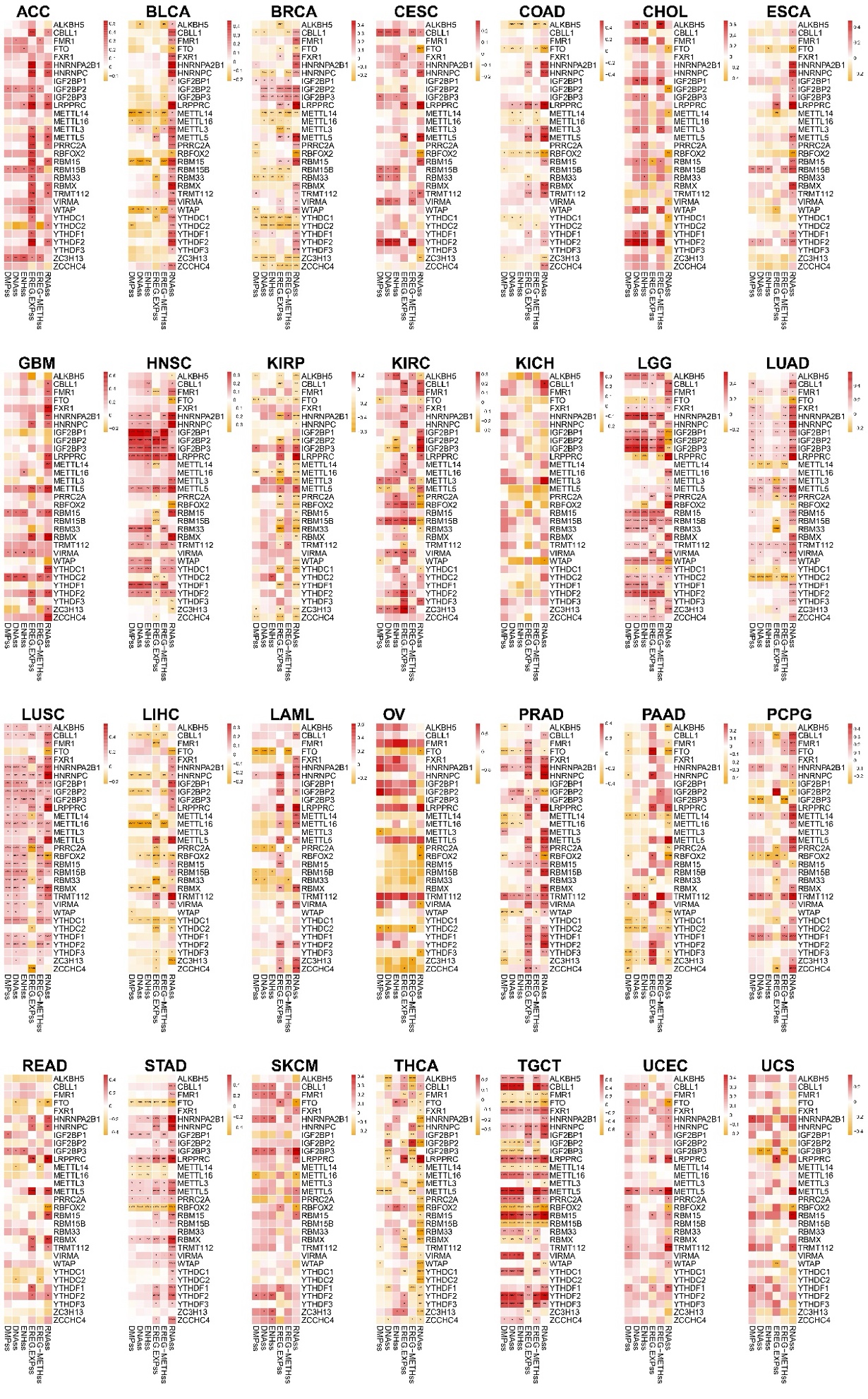


**Supplementary Figure S8. Correlation between m^6^A modification genes and tumor stemness scores in each cancer type.**


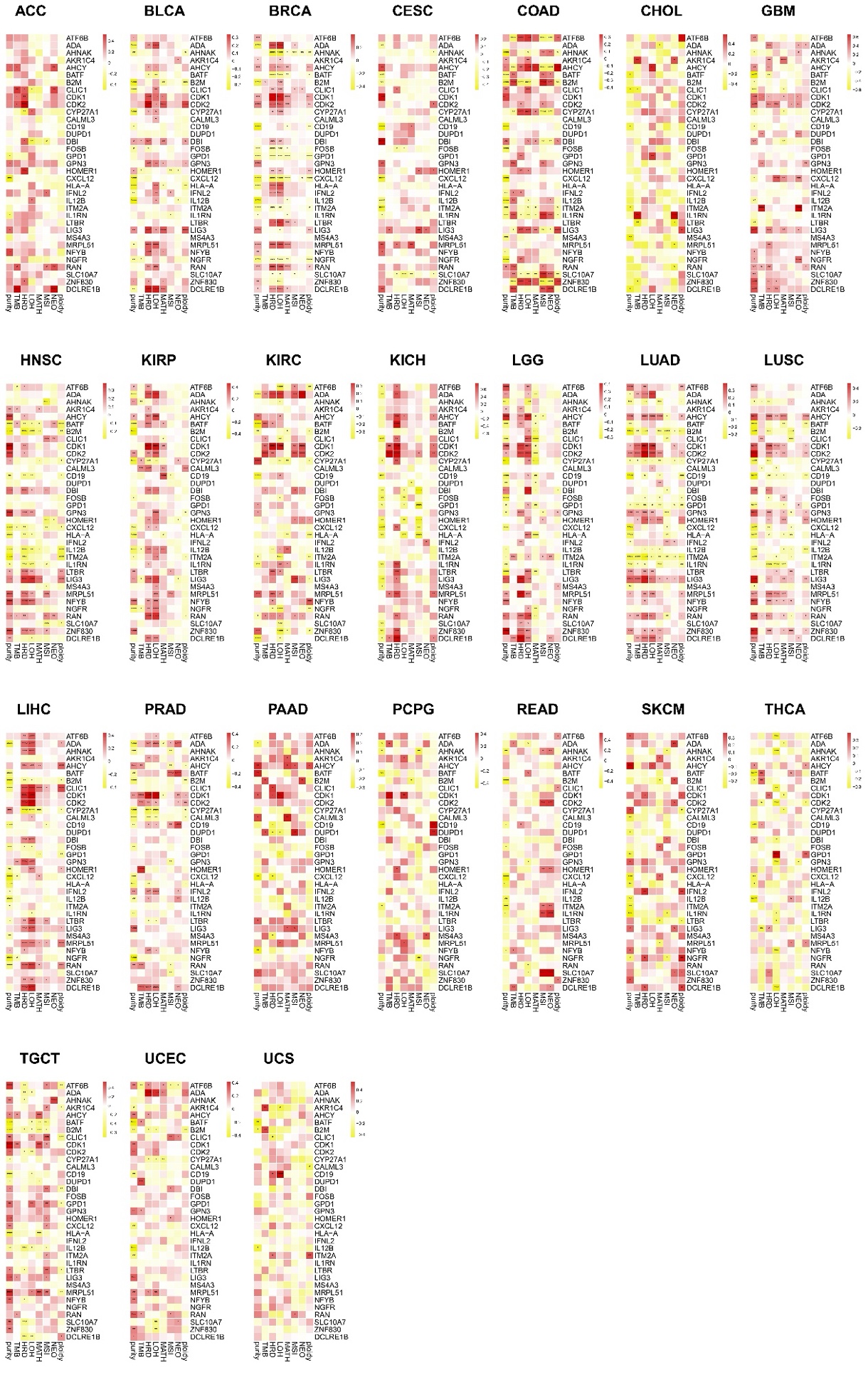


**Supplementary Figure S9. Correlation between m^6^A modification genes and genomic heterogeneity scores in each cancer type.**


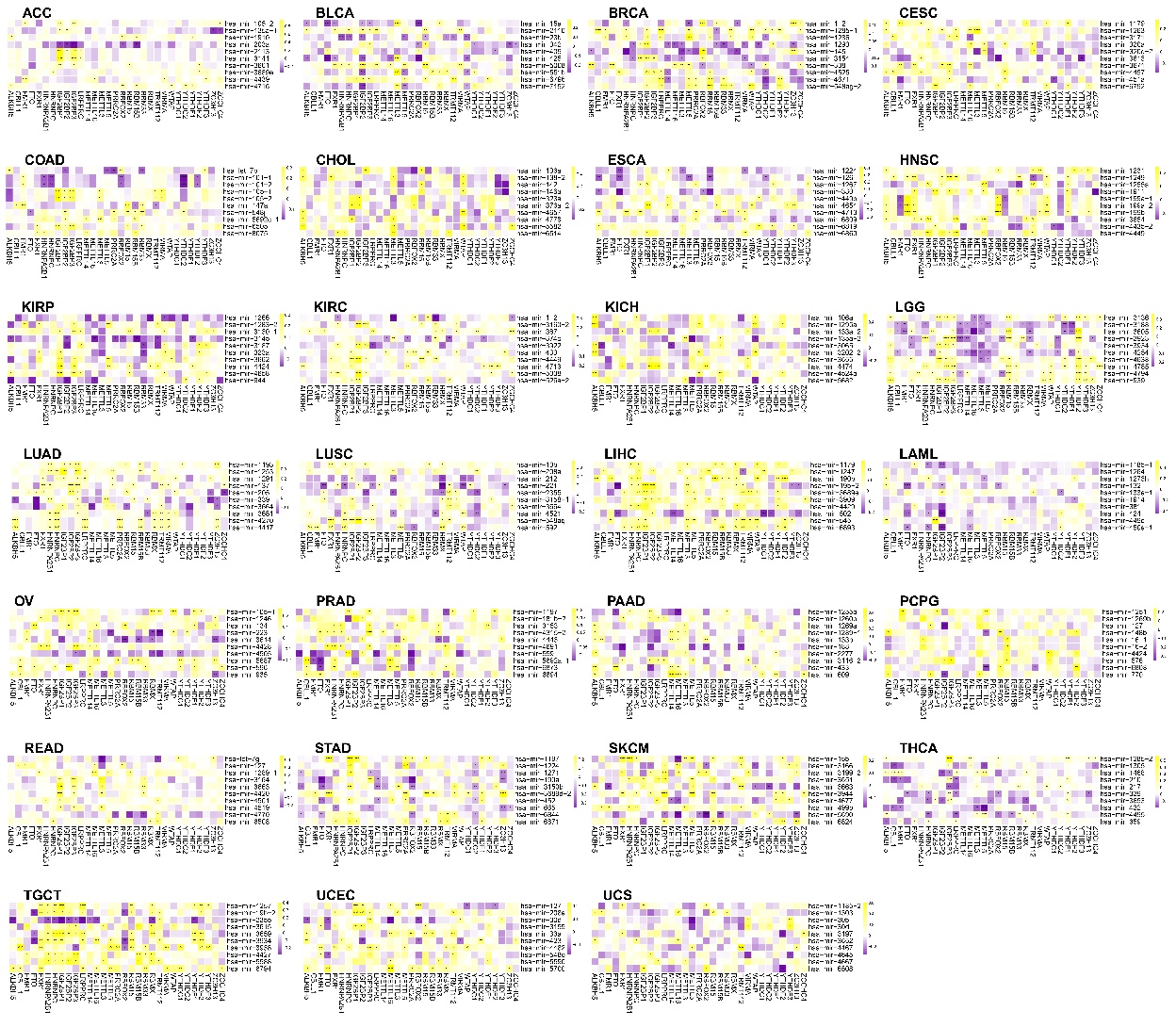


**Supplementary Figure S10. The relationship between m^6^A modification genes and miRNAs in pan-cancer.** (**p* < 0.05, ***p* < 0.01, ****p* < 0.001, *****p* < 0.0001).


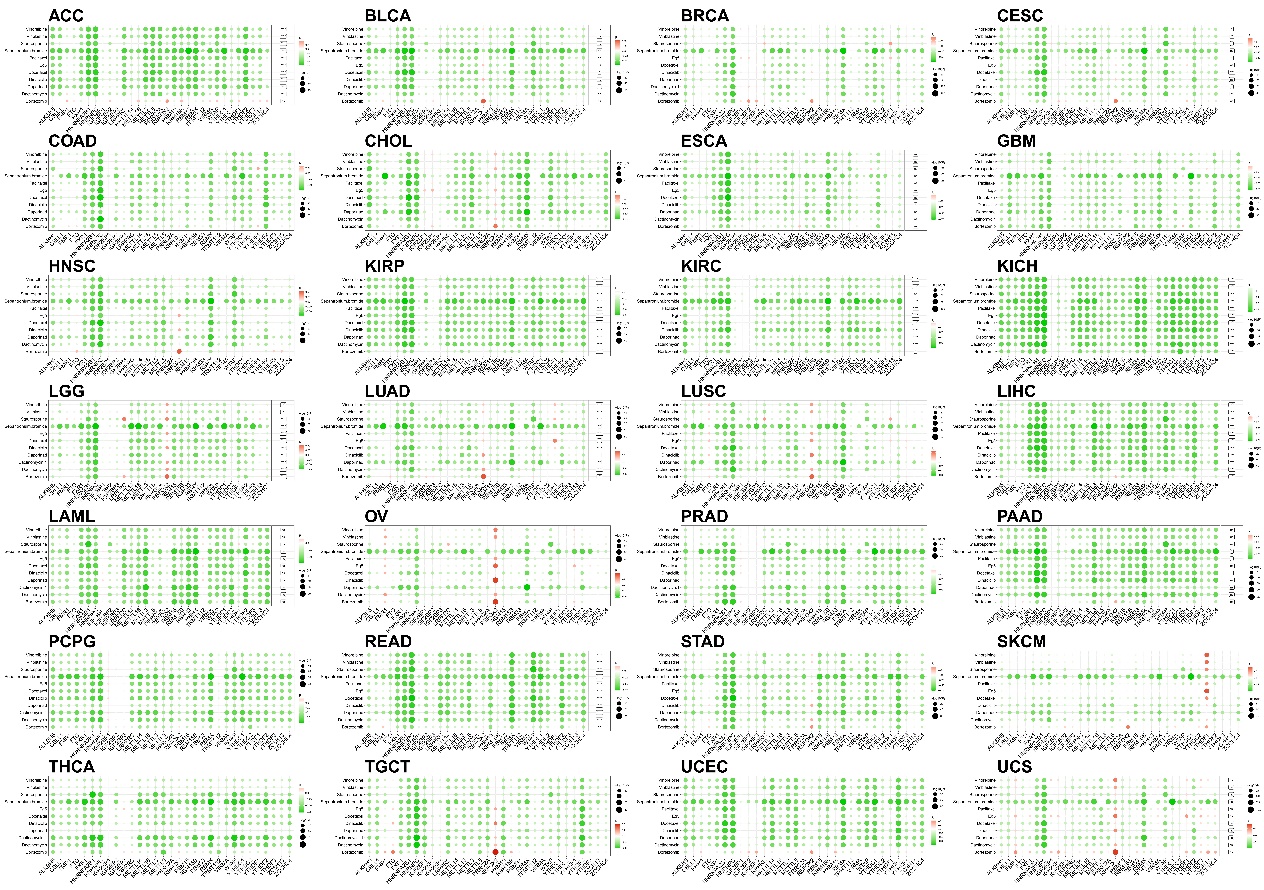


**Supplementary Figure S11. Drug prediction based on m^6^A modification genes.** Relationship between top 10 predicted drugs and genes in each cancer. Differential drugs between high- and low-risk groups in each tumor were shown.
