# Supplementary figures and images for: Interplay of RNA m6A Modification-Related Geneset in Pan-Cancer
Source: Biomedicines. 2024 Sep 27;12(10):2211. doi: 10.3390/biomedicines12102211 (PMC11504890; doi:10.3390/biomedicines12102211)

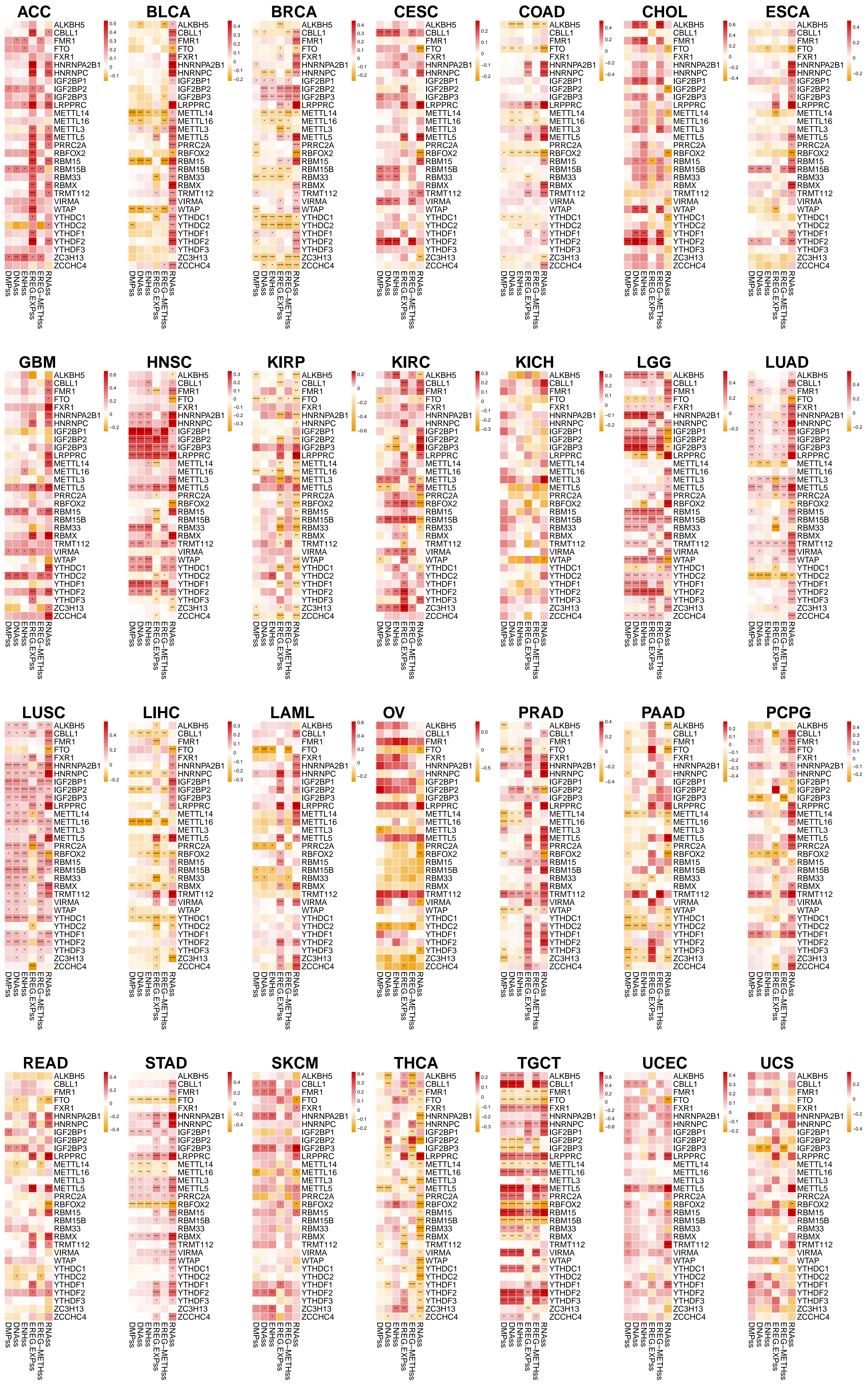

Supplement: Supplementary file 1 [file biomedicines-12-02211-s001.zip › Supplementary Files/Supplementary Figure 8.pdf]

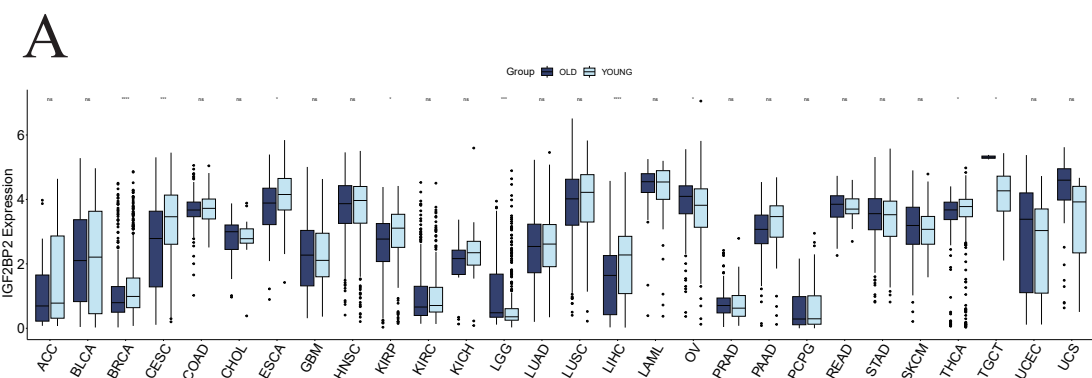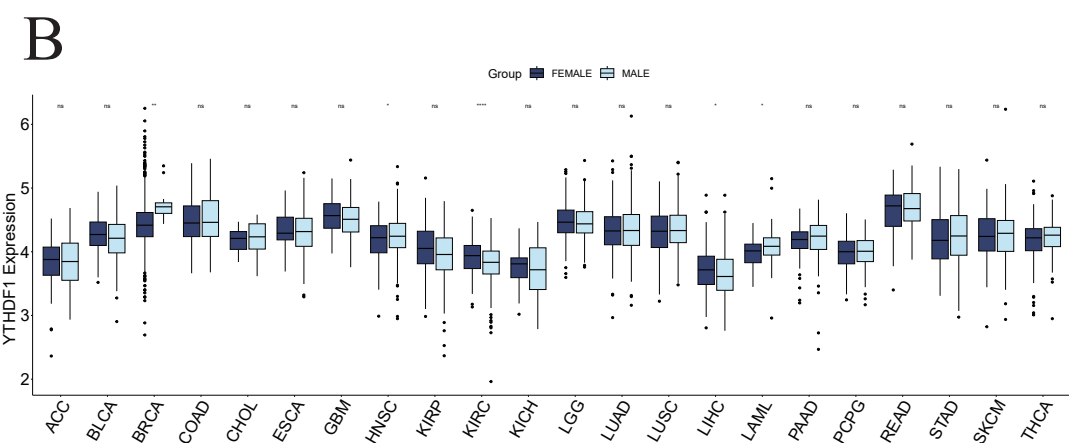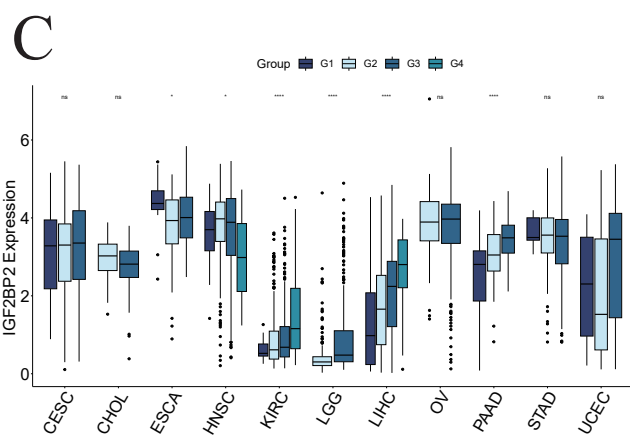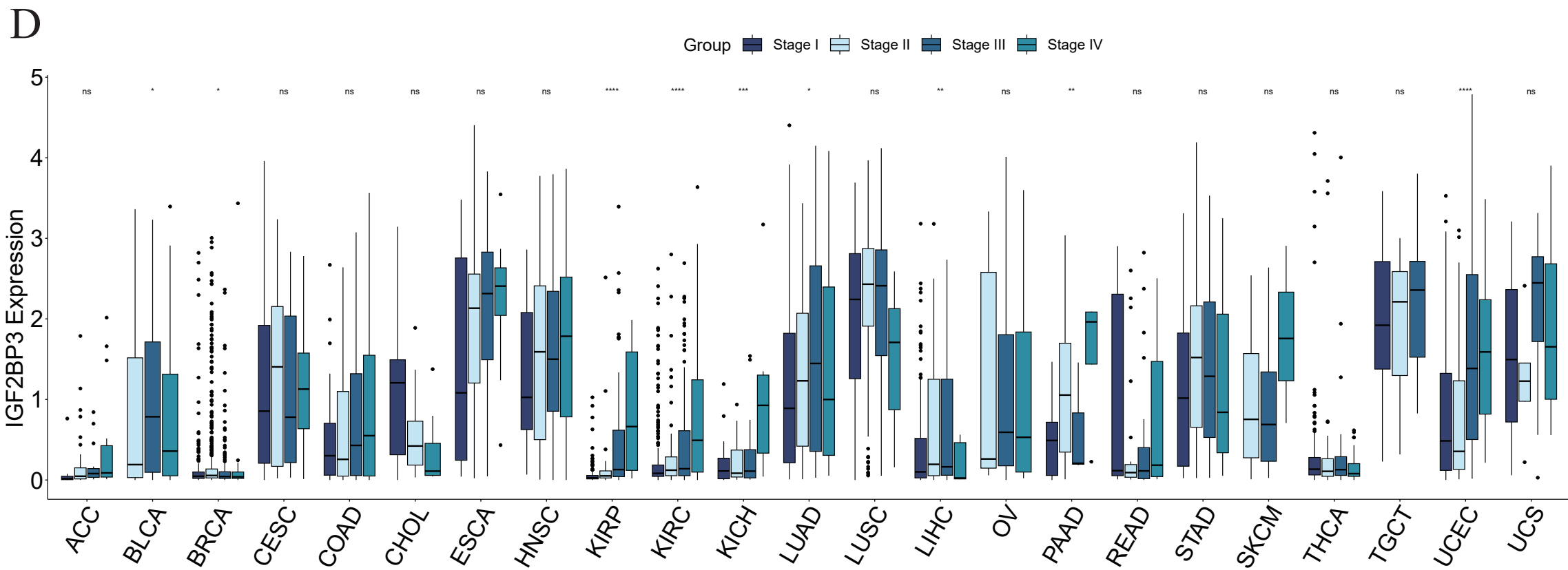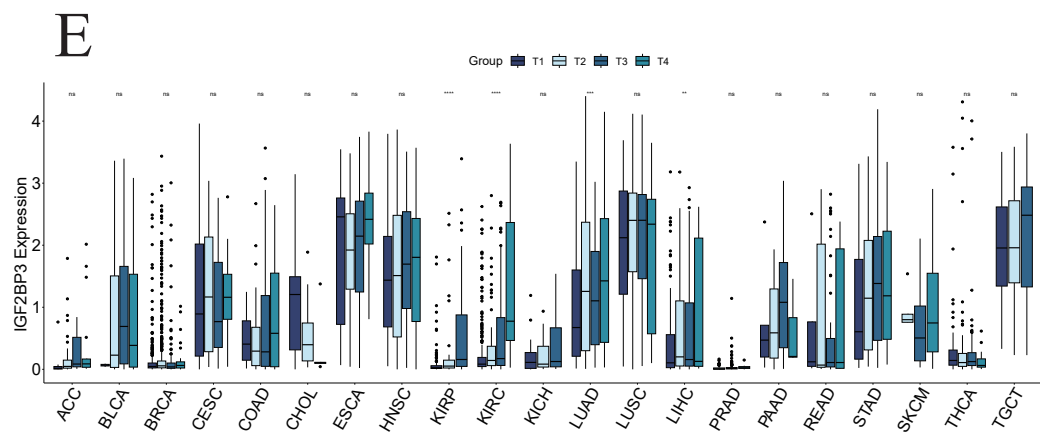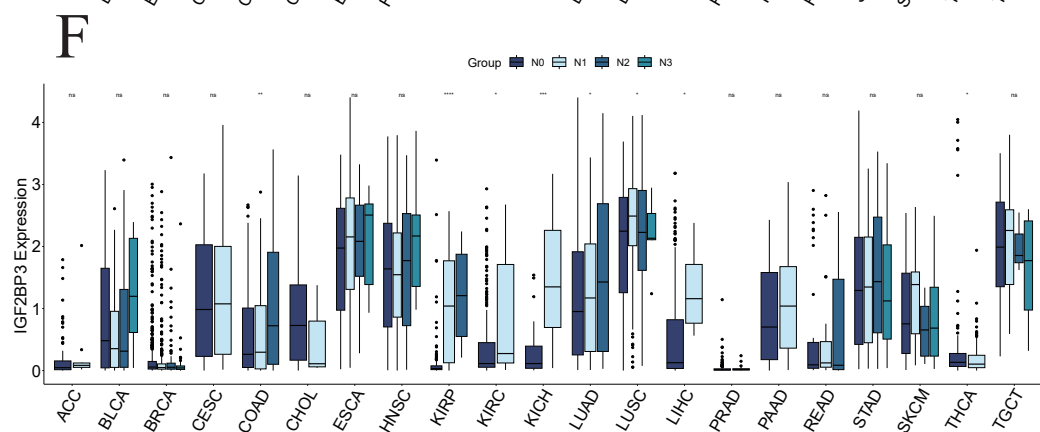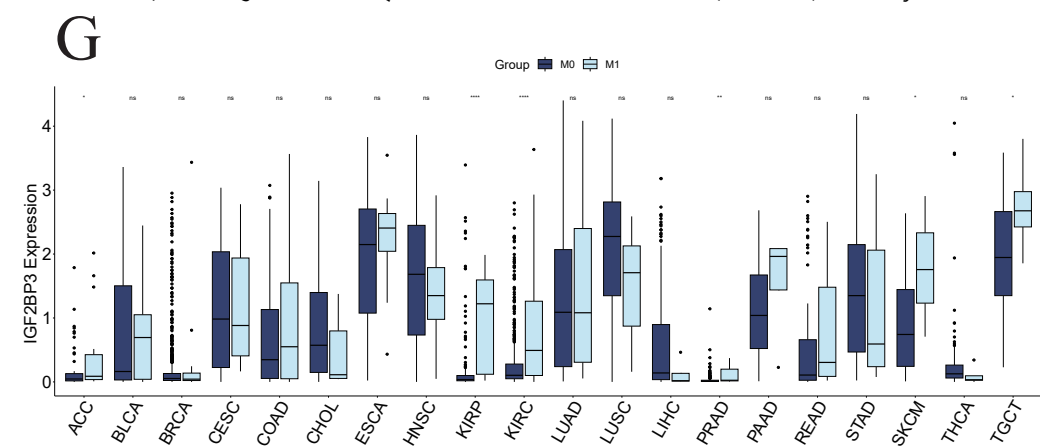

Supplement: Supplementary file 1 [file biomedicines-12-02211-s001.zip › Supplementary Files/Supplementary Figure 4.pdf]

A

CNV percentage in each cancer

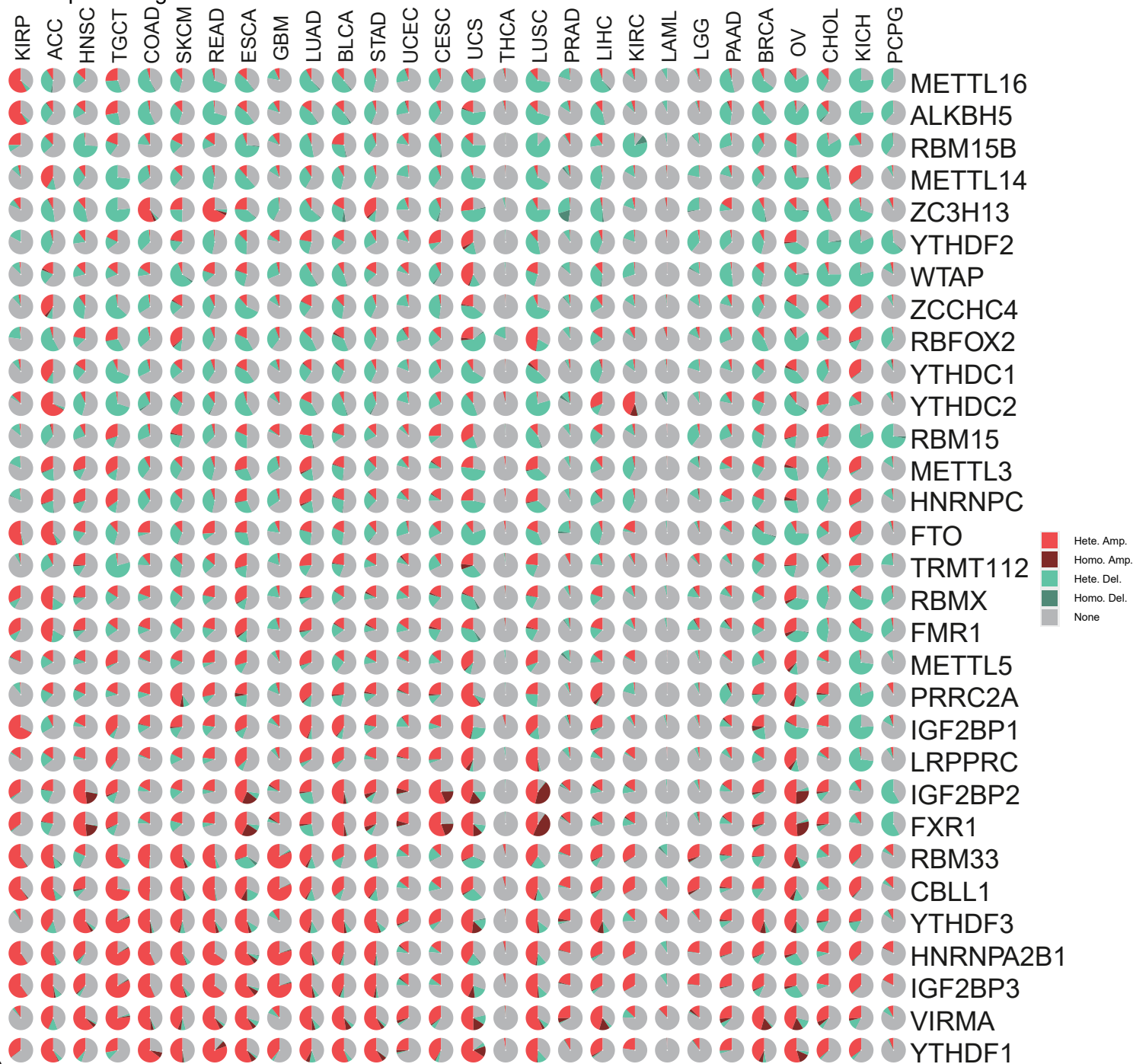

B

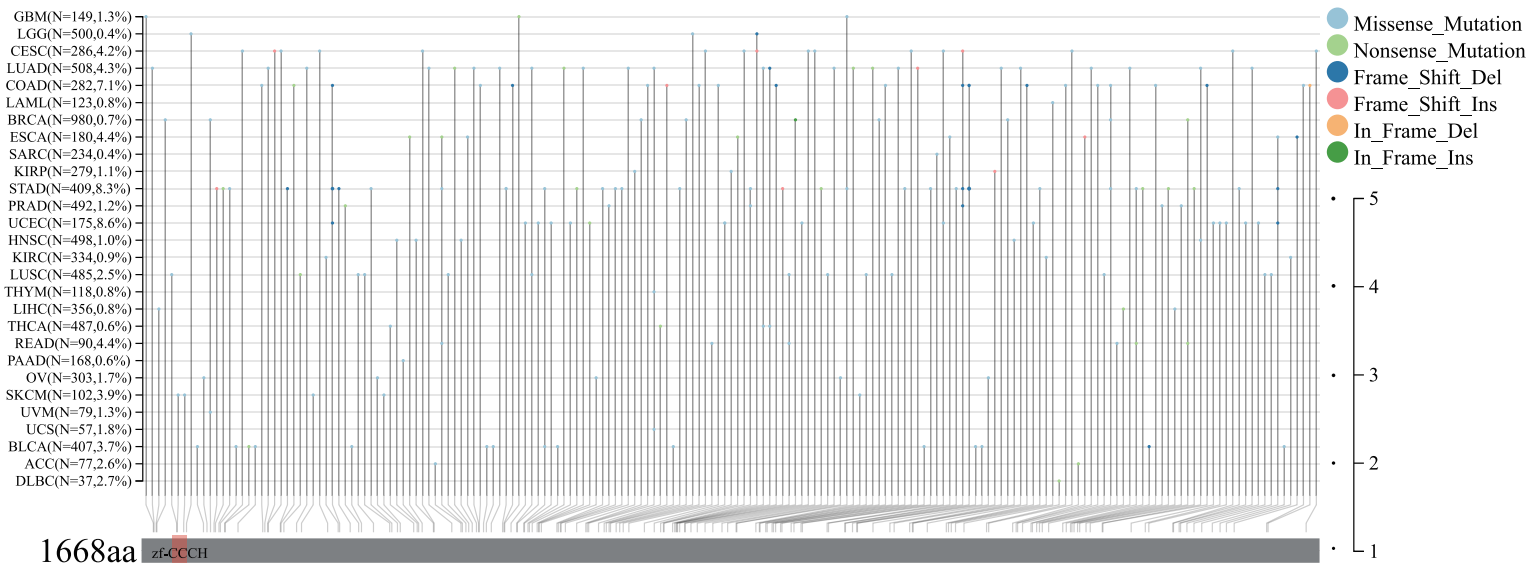

Supplement: Supplementary file 1 [file biomedicines-12-02211-s001.zip › Supplementary Files/Supplementary Figure 5.pdf]

A

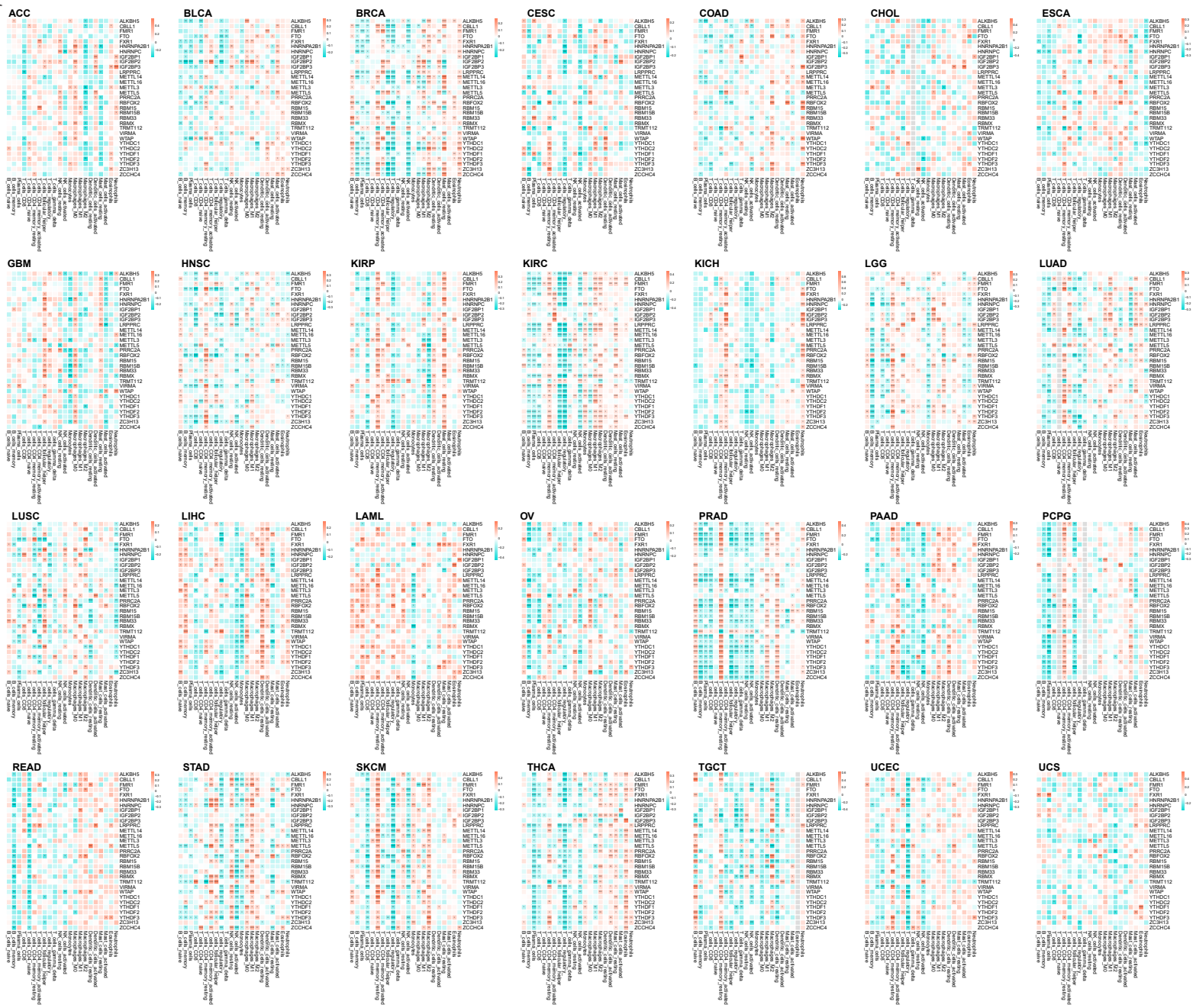

B

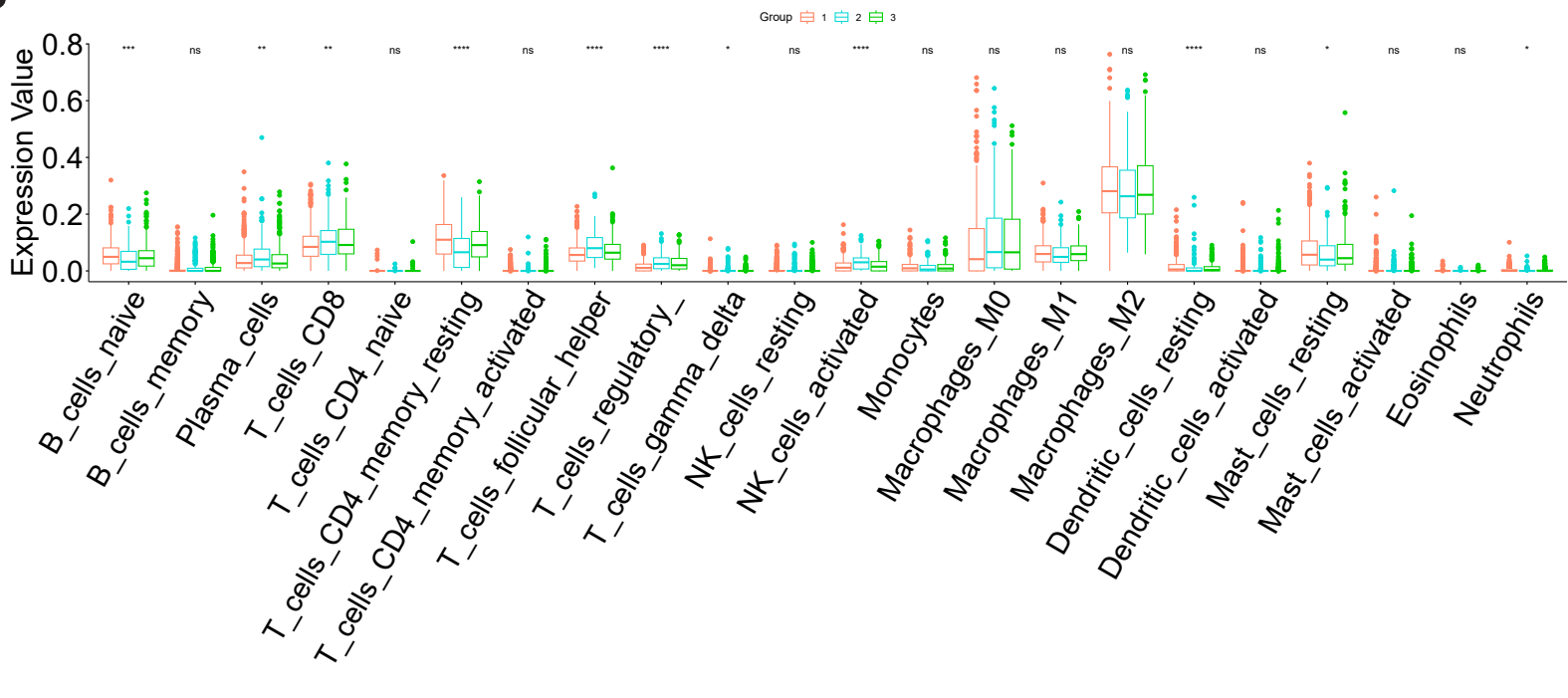

Supplement: Supplementary file 1 [file biomedicines-12-02211-s001.zip › Supplementary Files/Supplementary Figure 7.pdf]

A

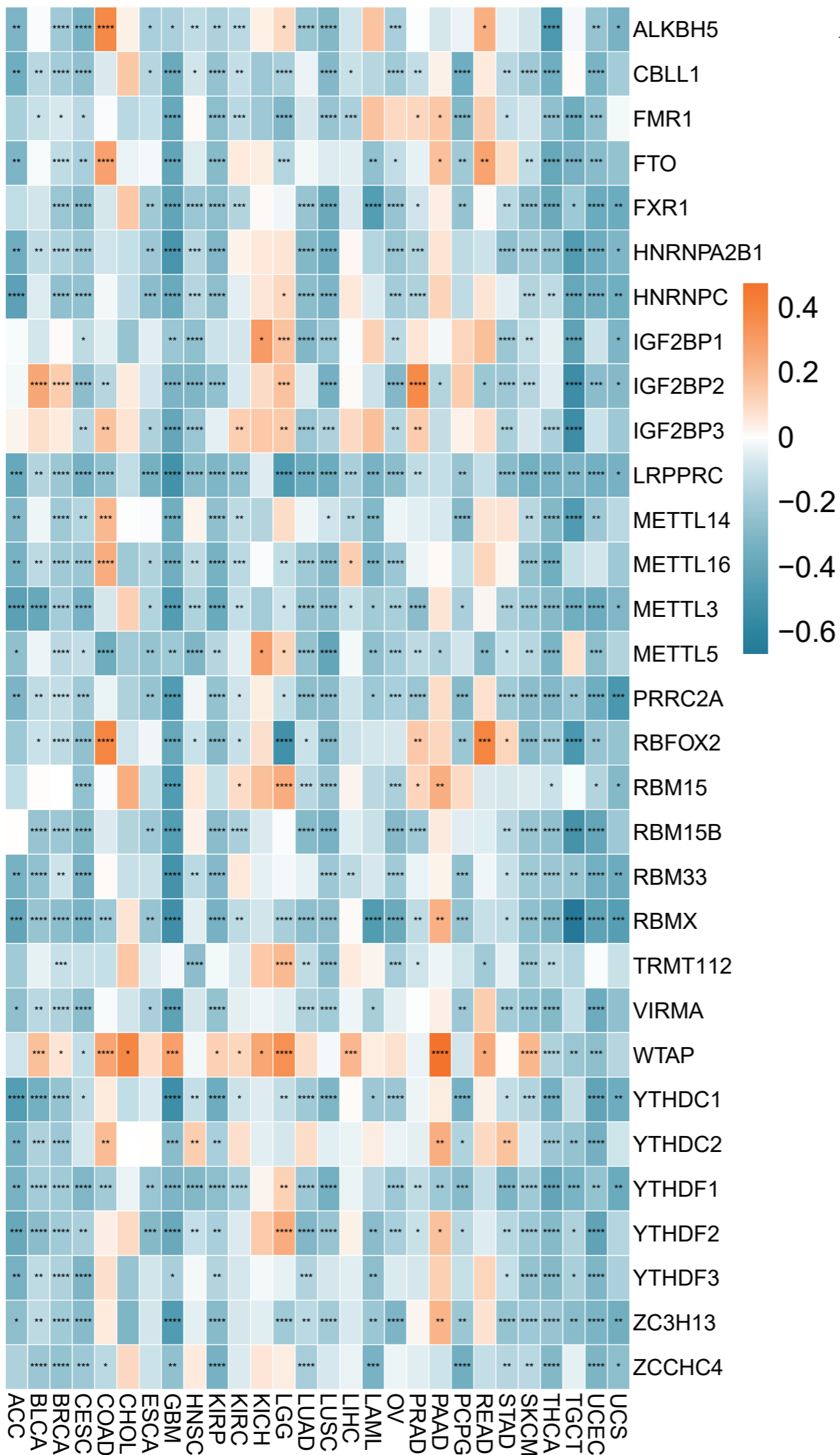

B

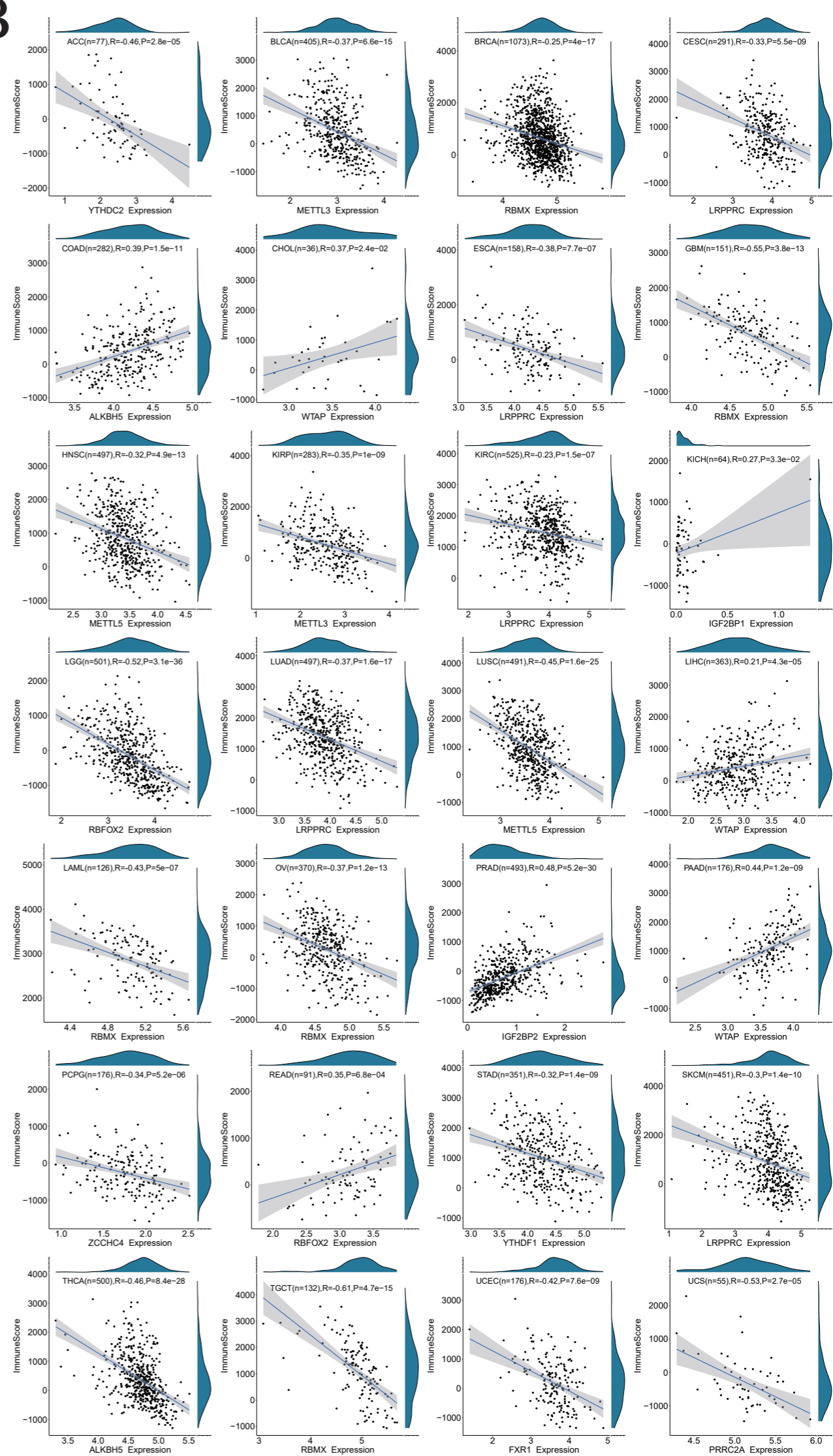

Supplement: Supplementary file 1 [file biomedicines-12-02211-s001.zip › Supplementary Files/Supplementary Figure 6.pdf]

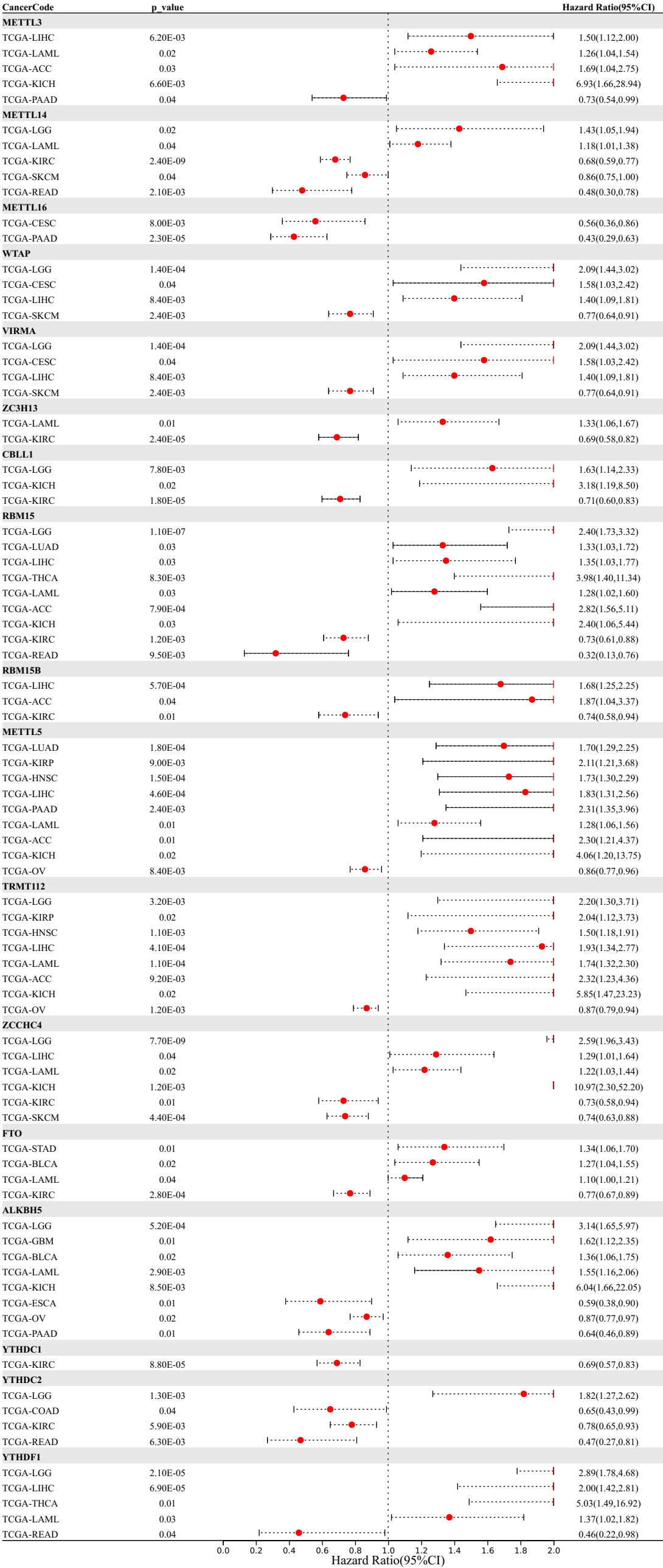

Supplement: Supplementary file 1 [file biomedicines-12-02211-s001.zip › Supplementary Files/Supplementary Figure 3.pdf]

A

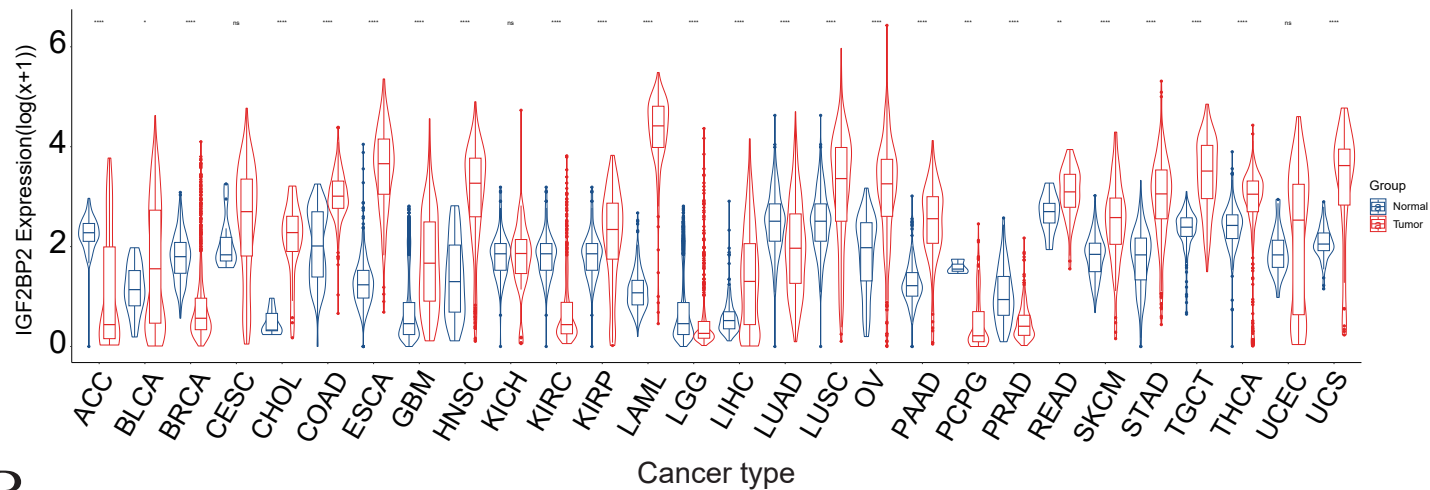

B

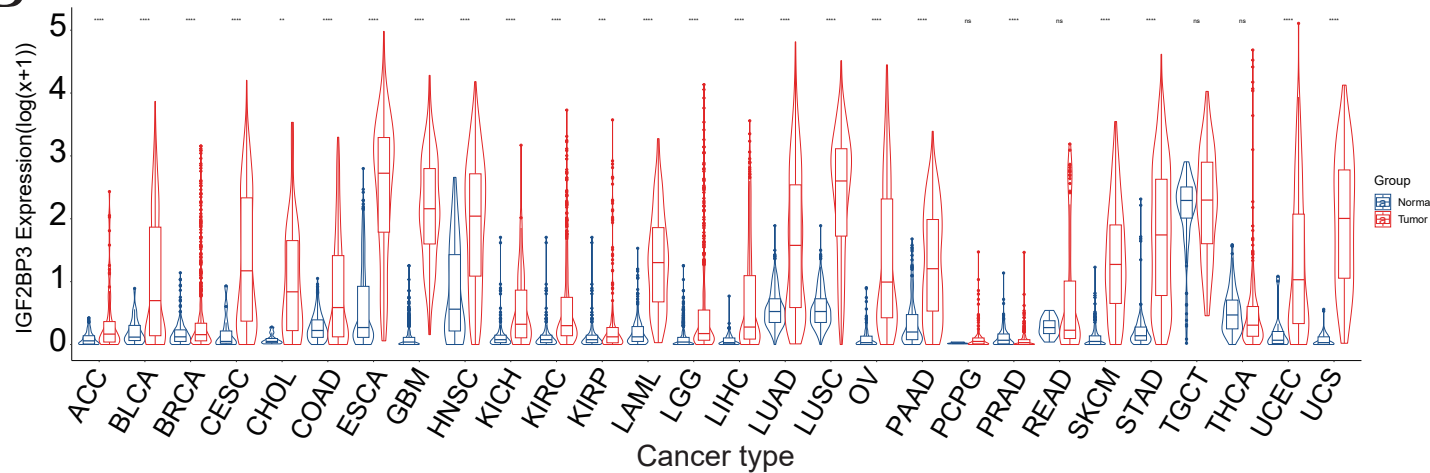

Supplement: Supplementary file 1 [file biomedicines-12-02211-s001.zip › Supplementary Files/Supplementary Figure 1.pdf]

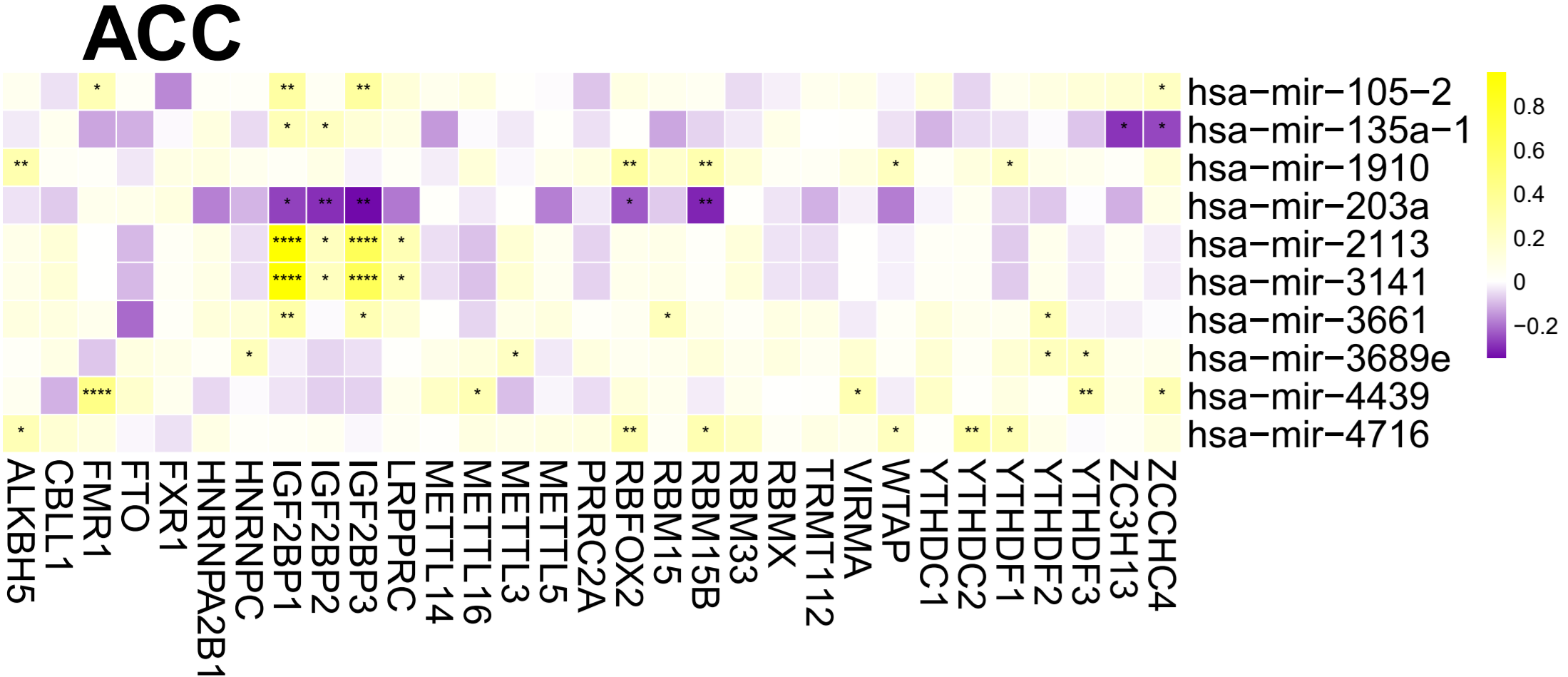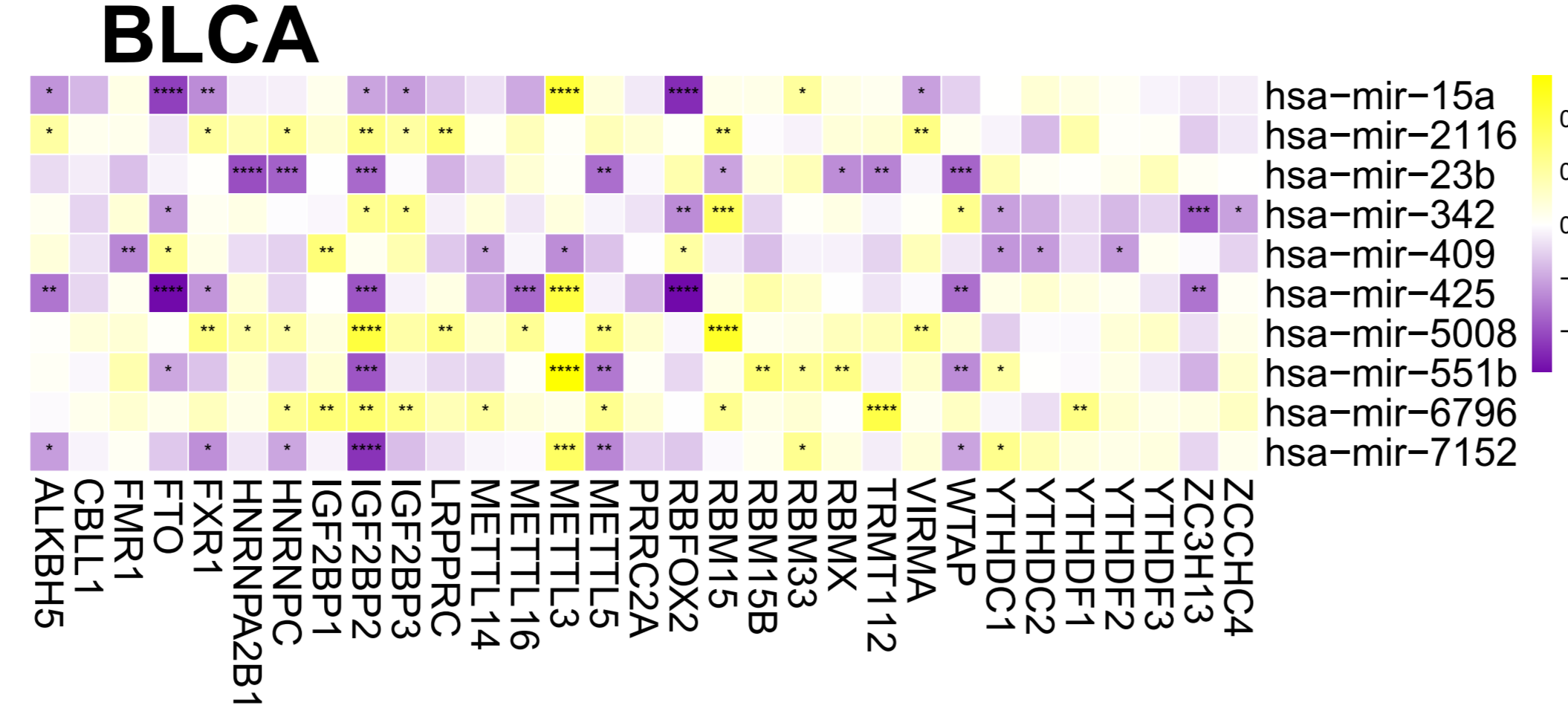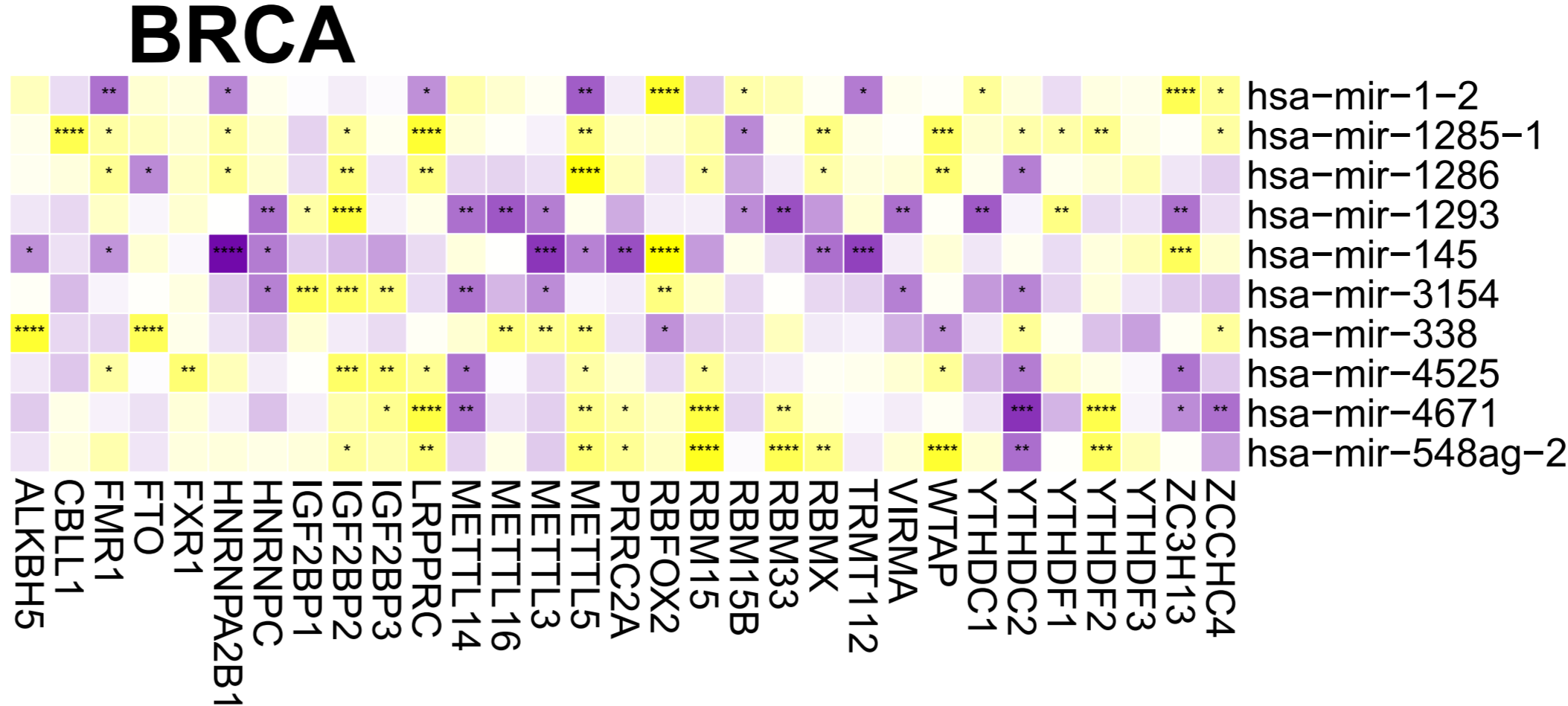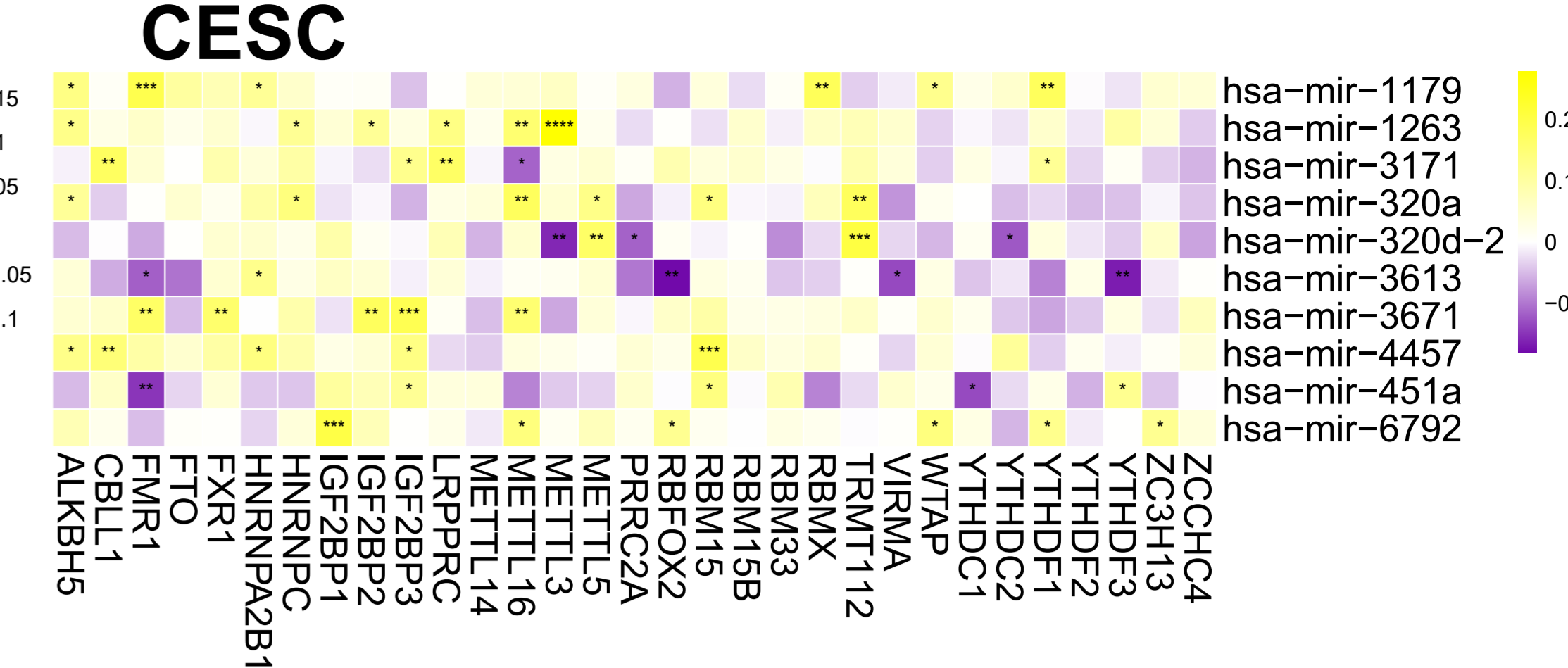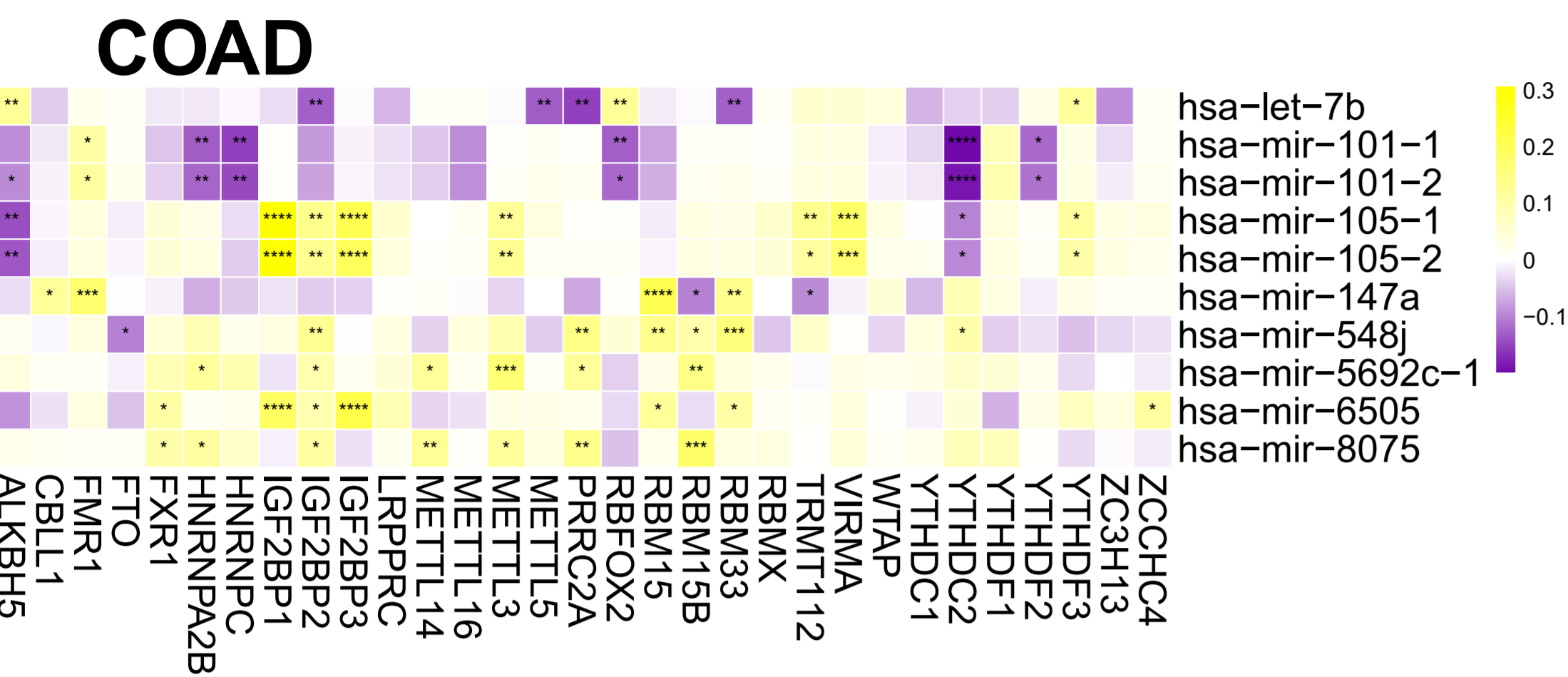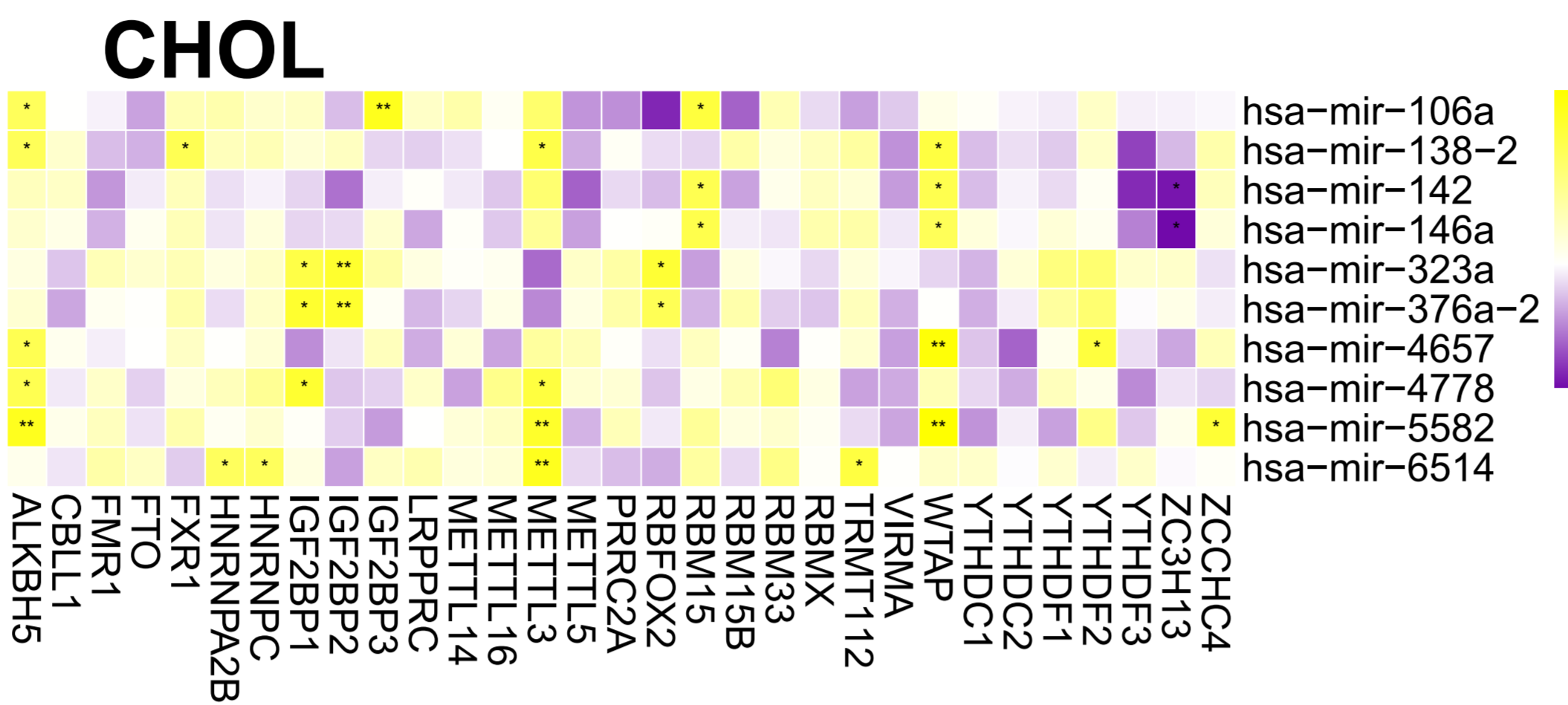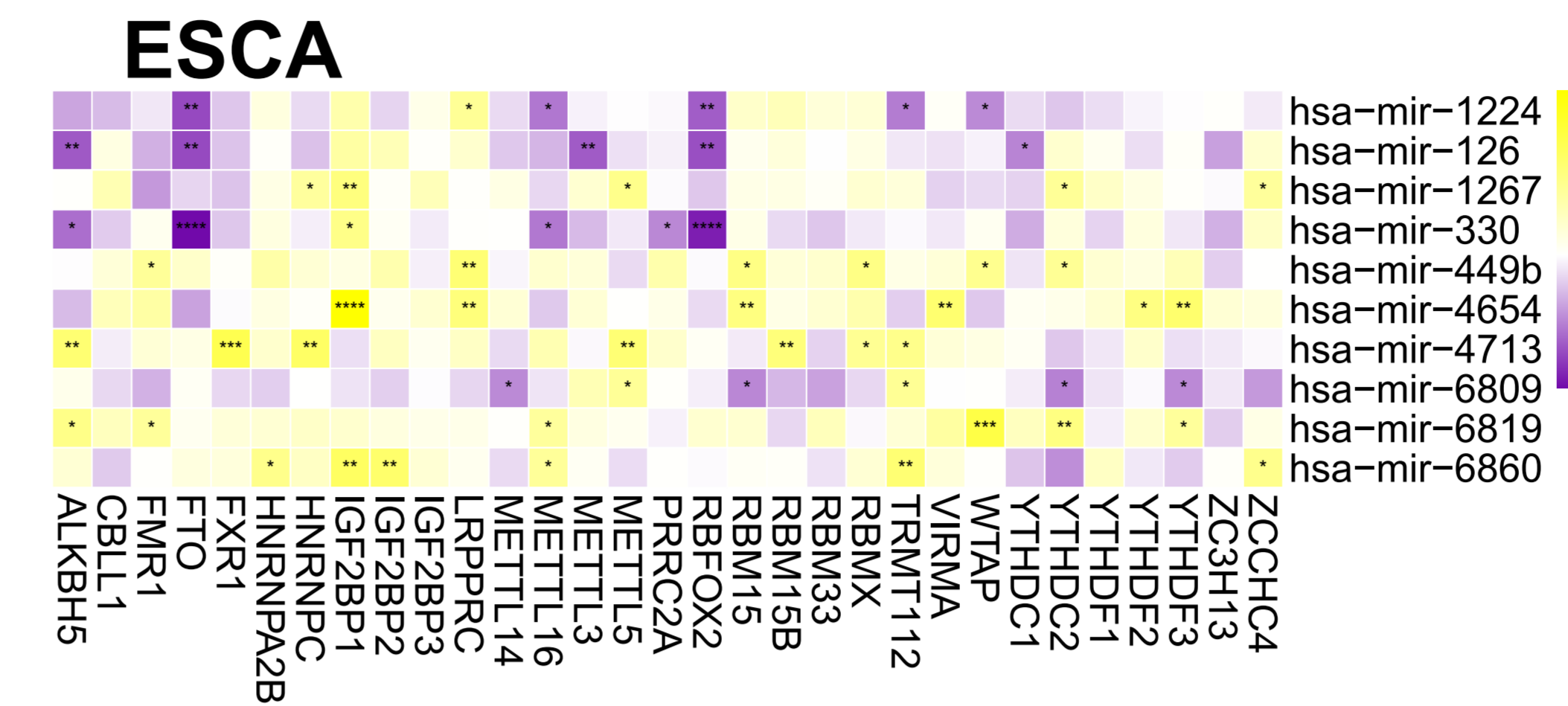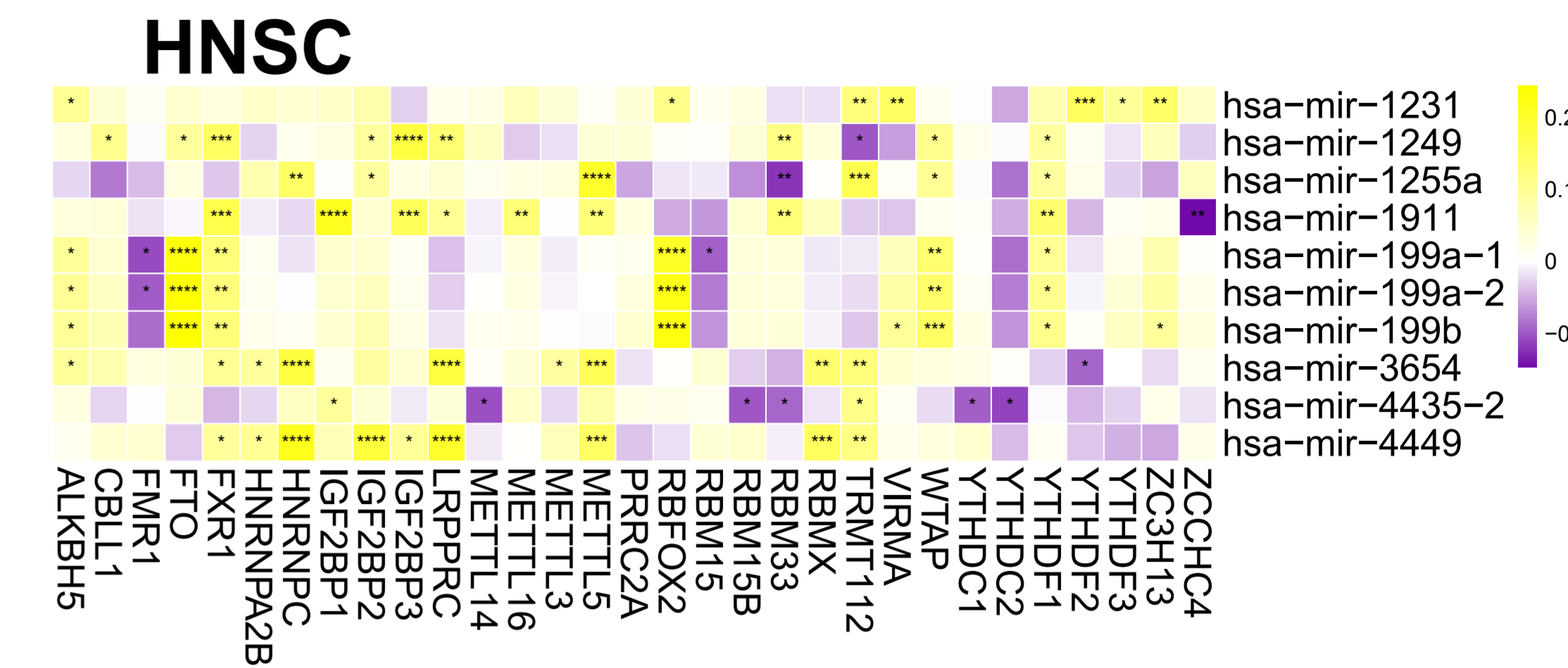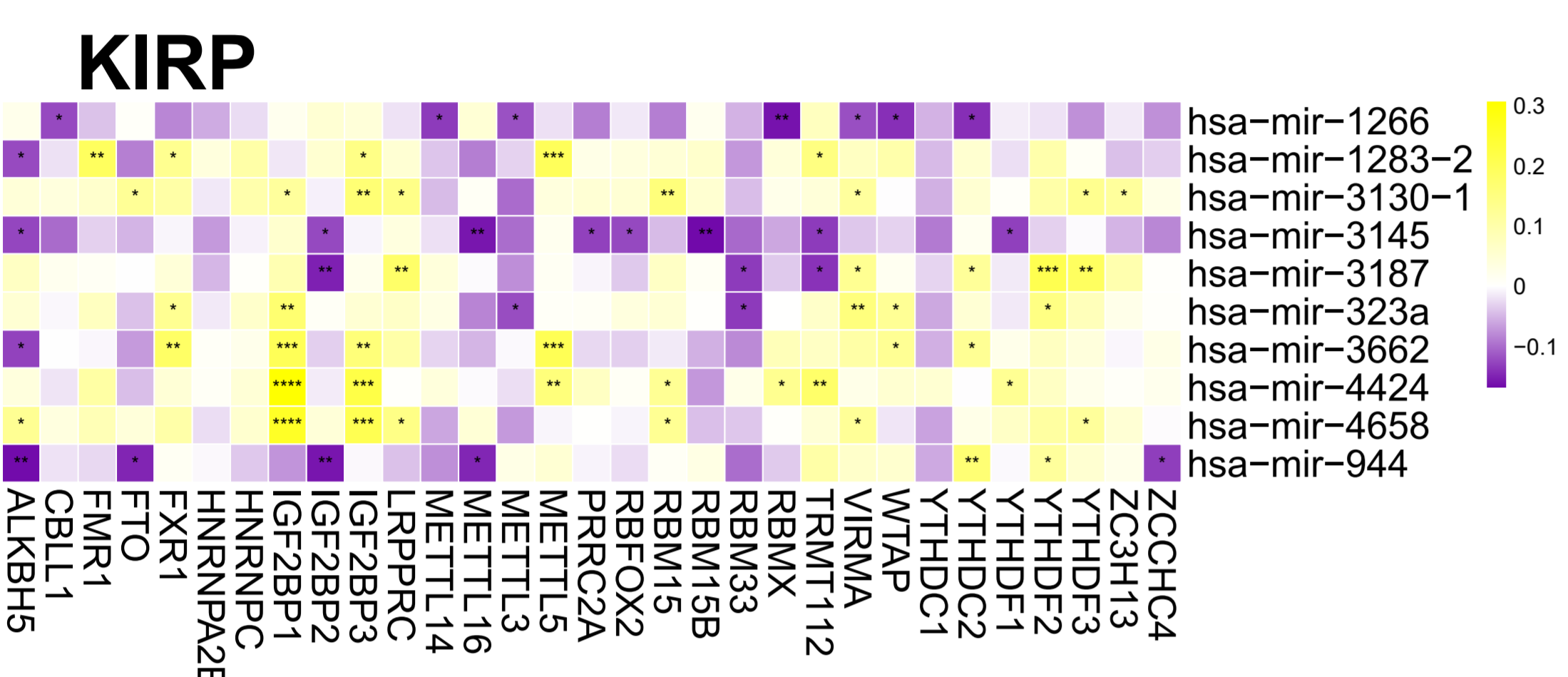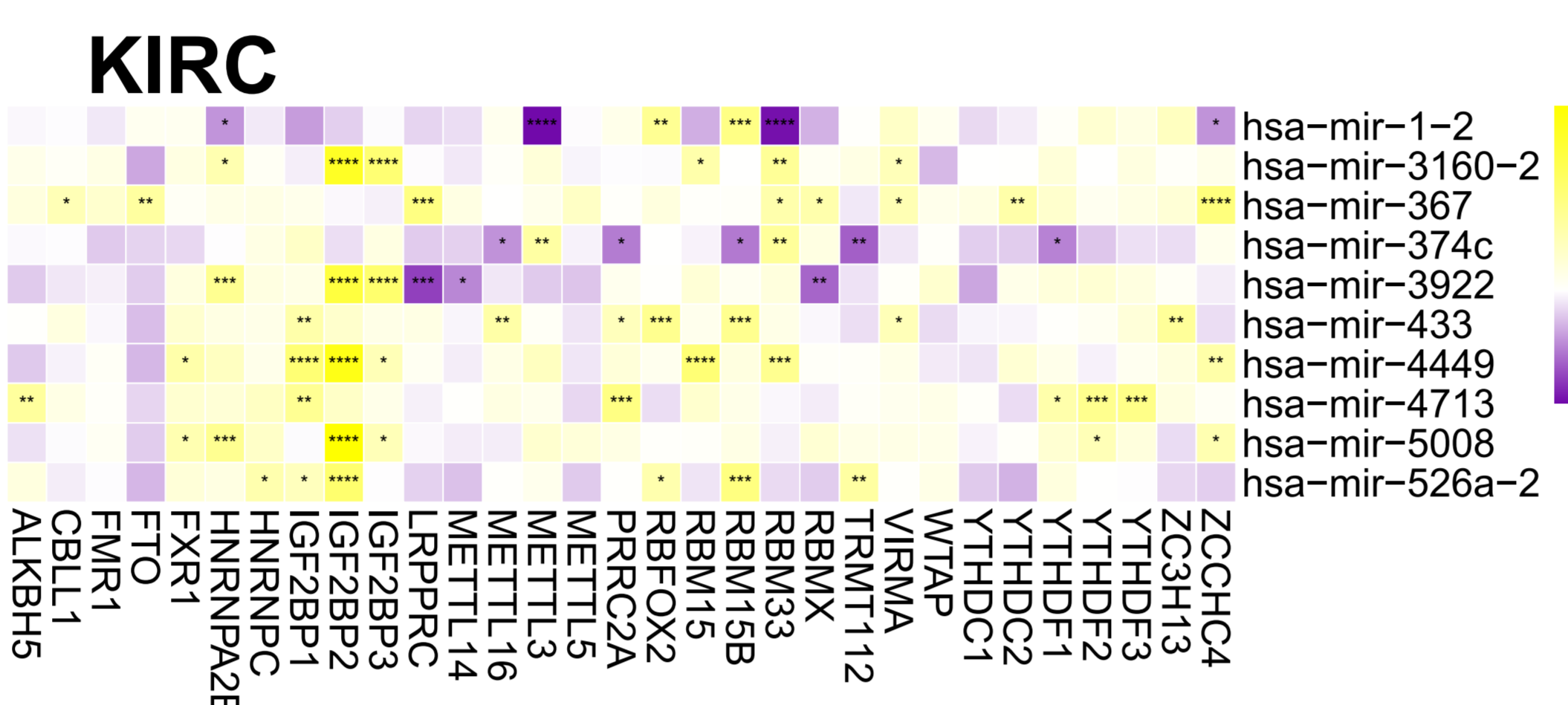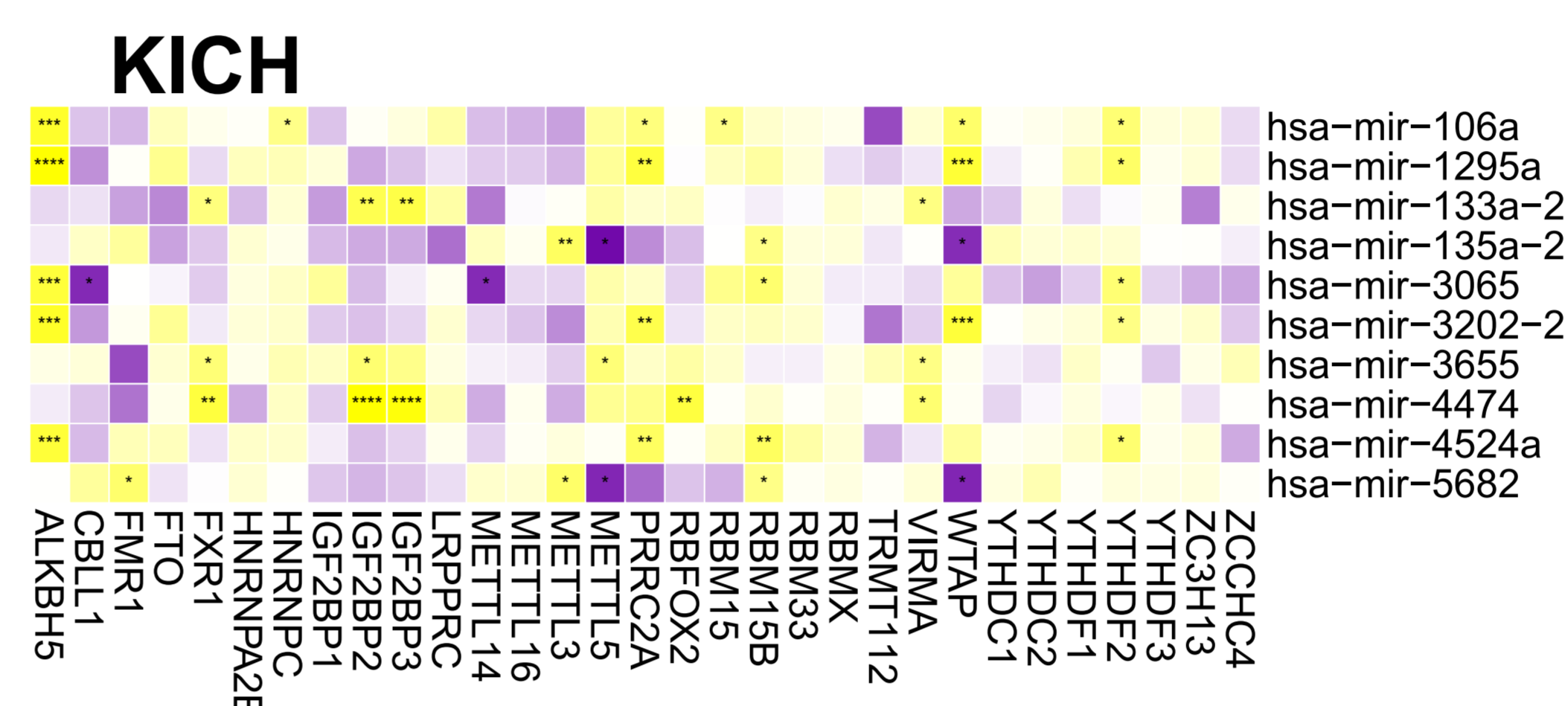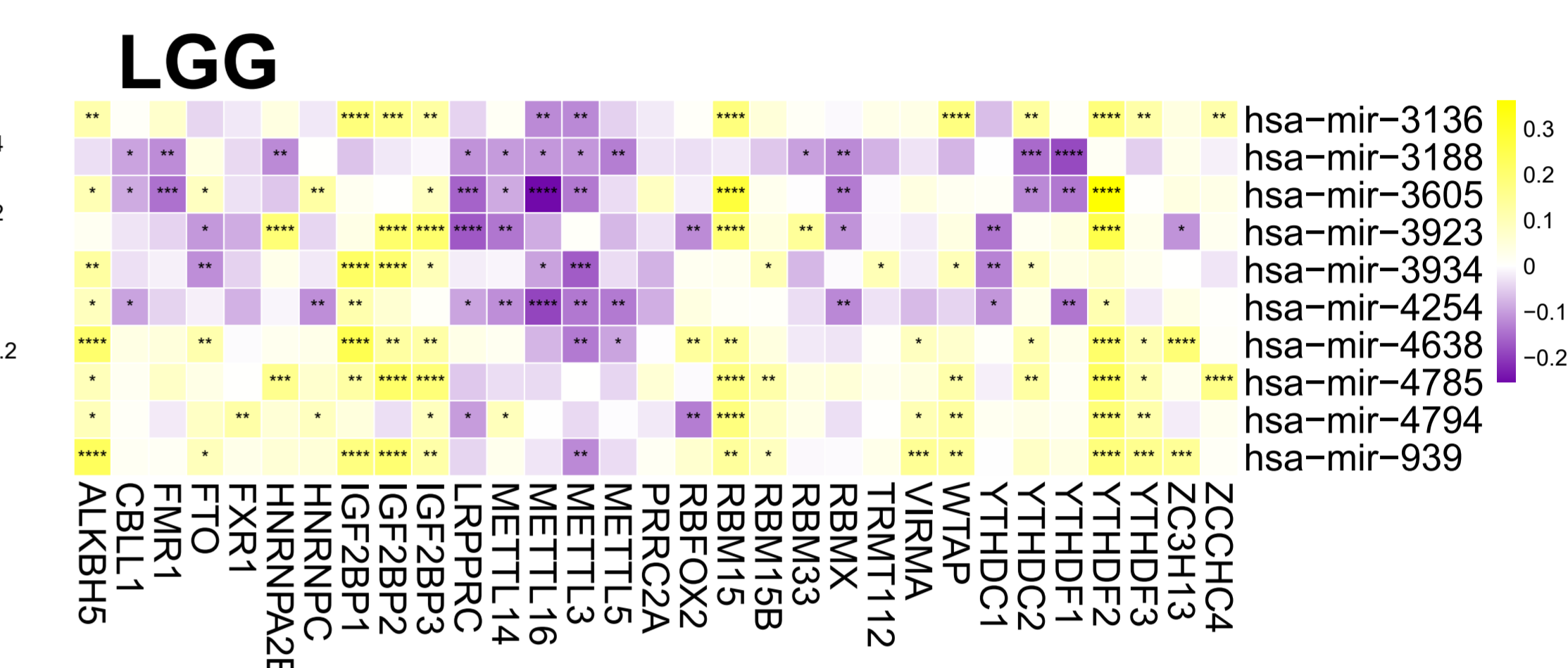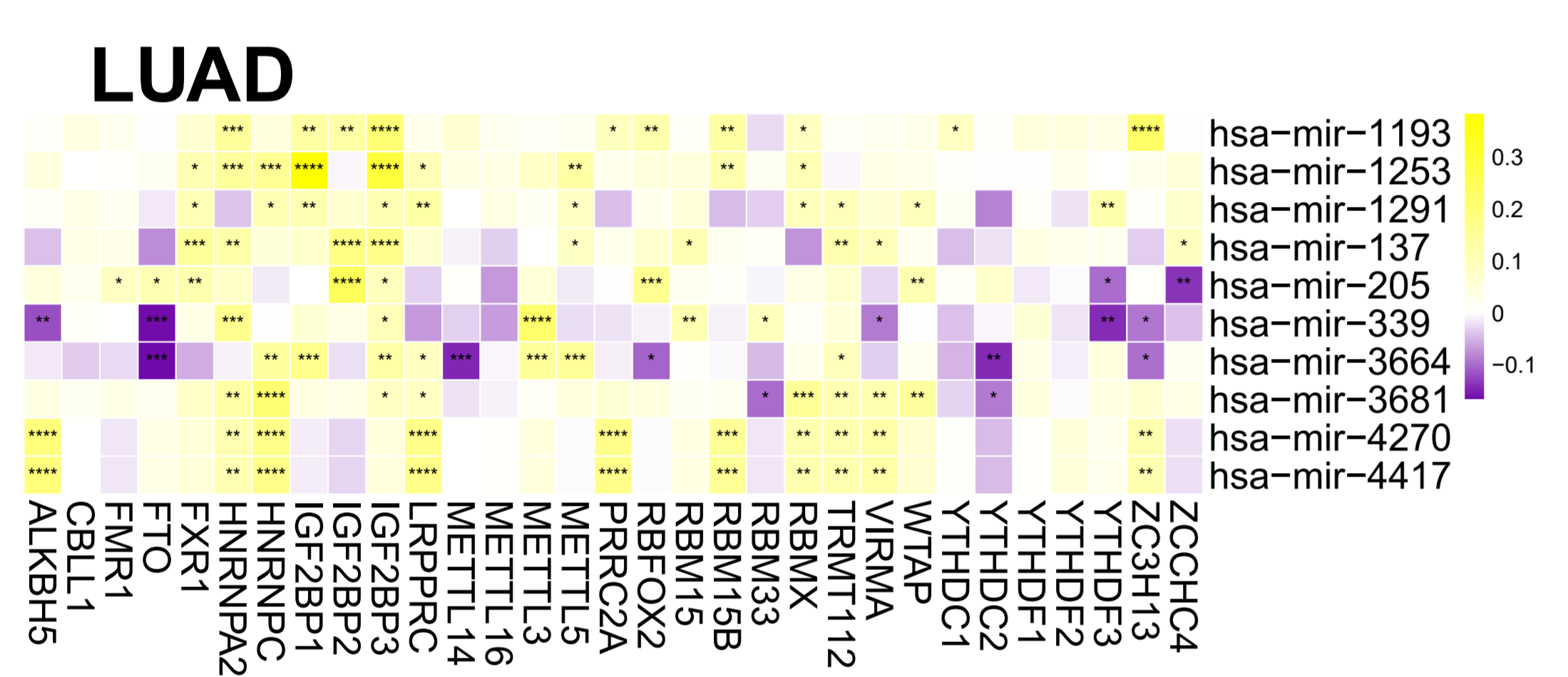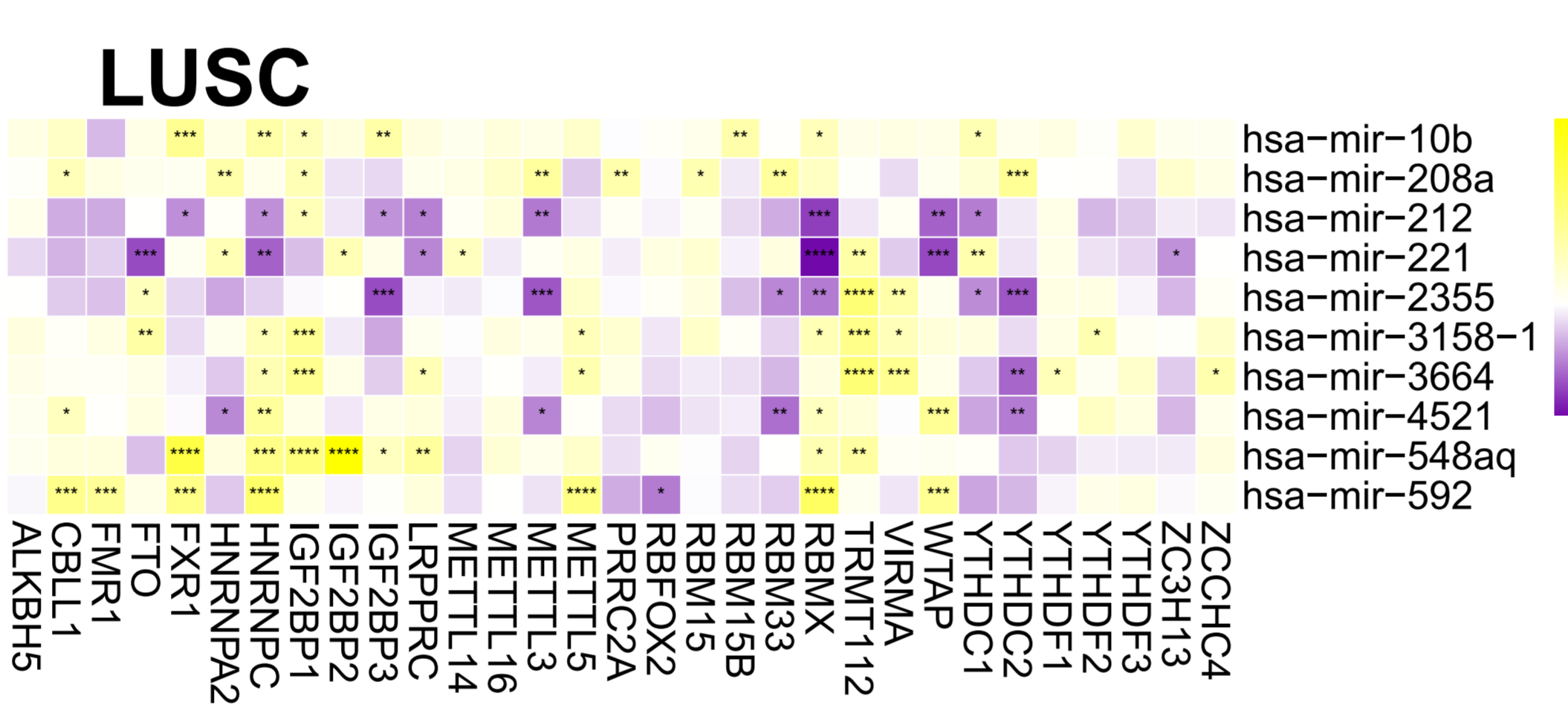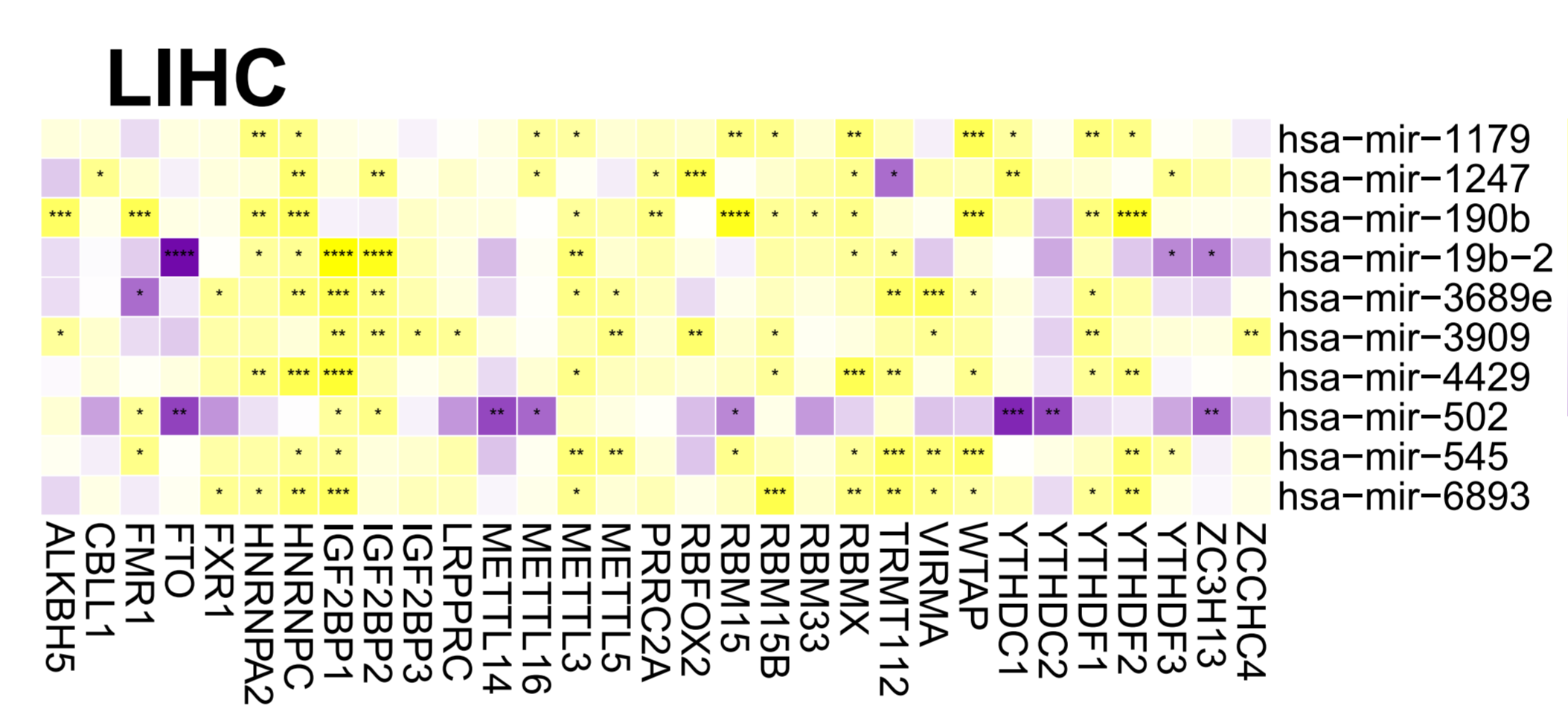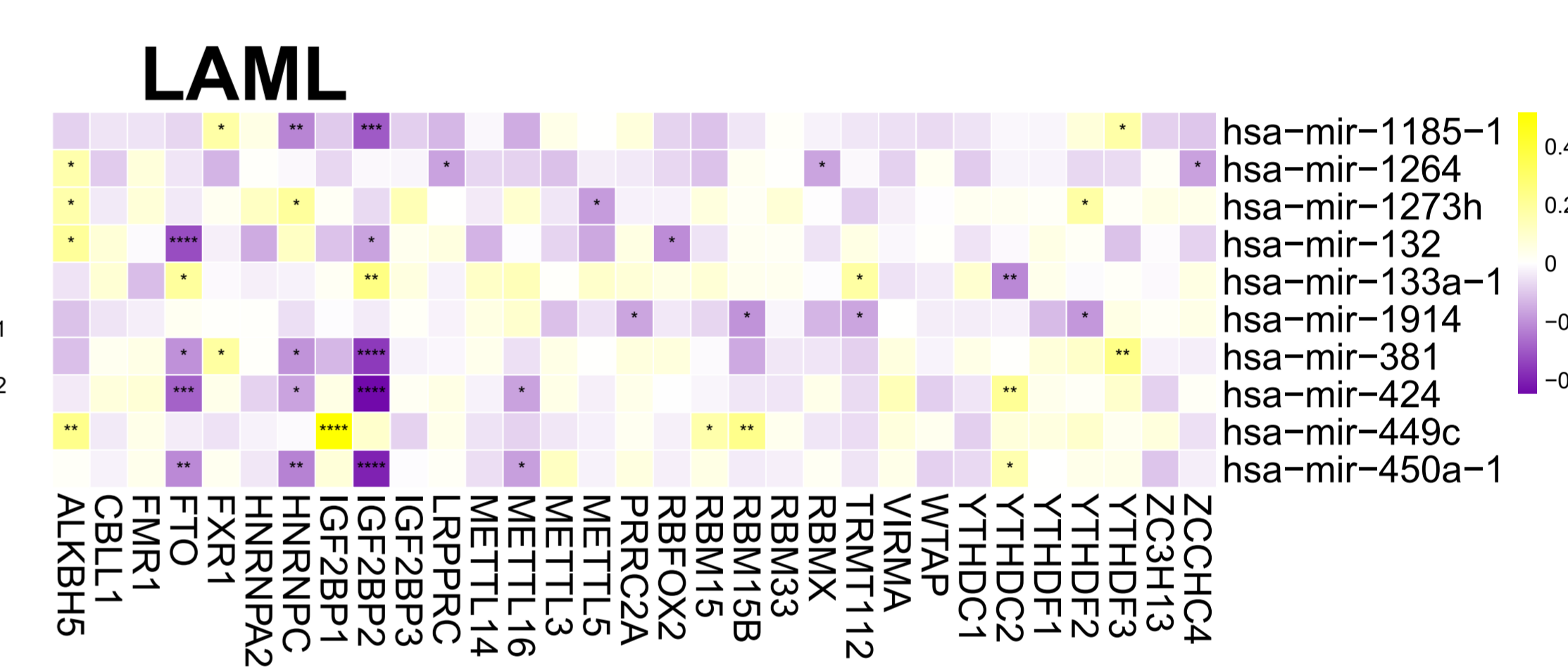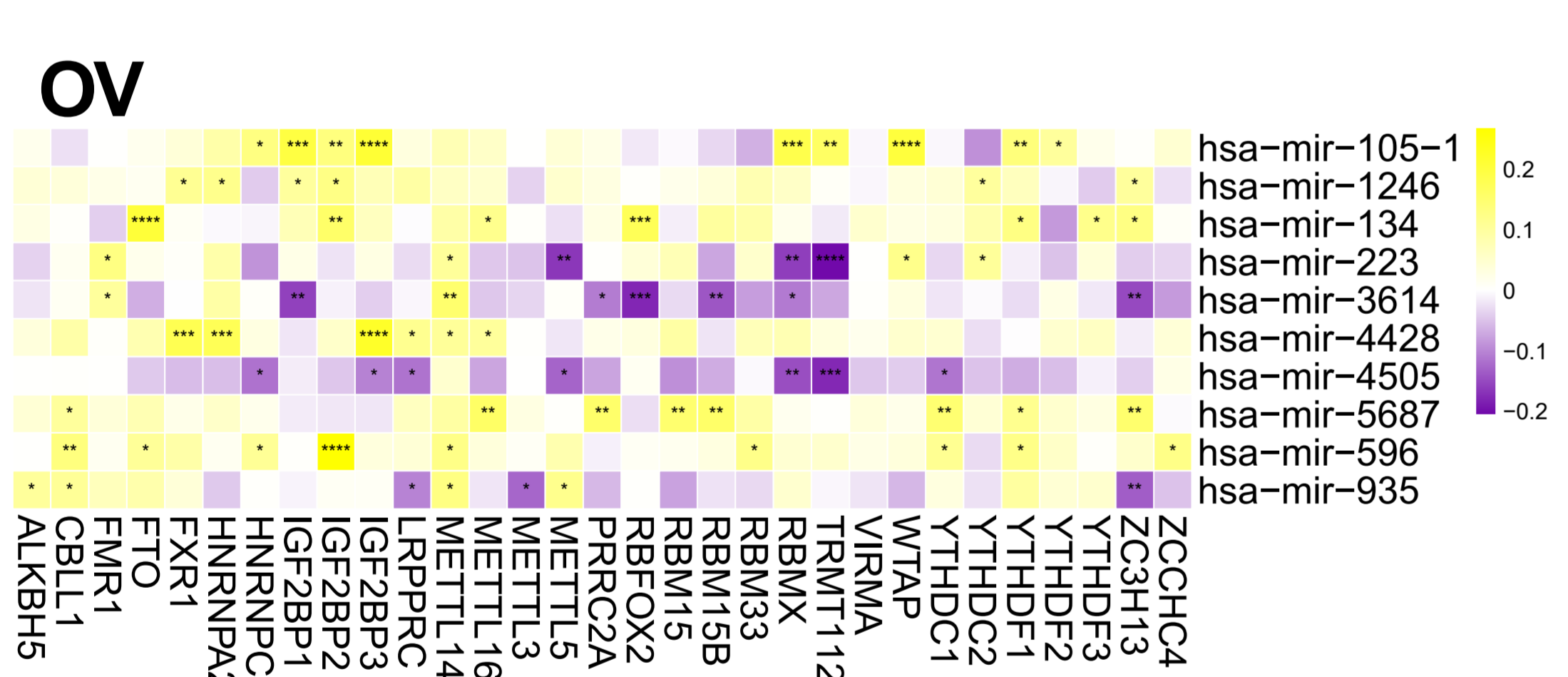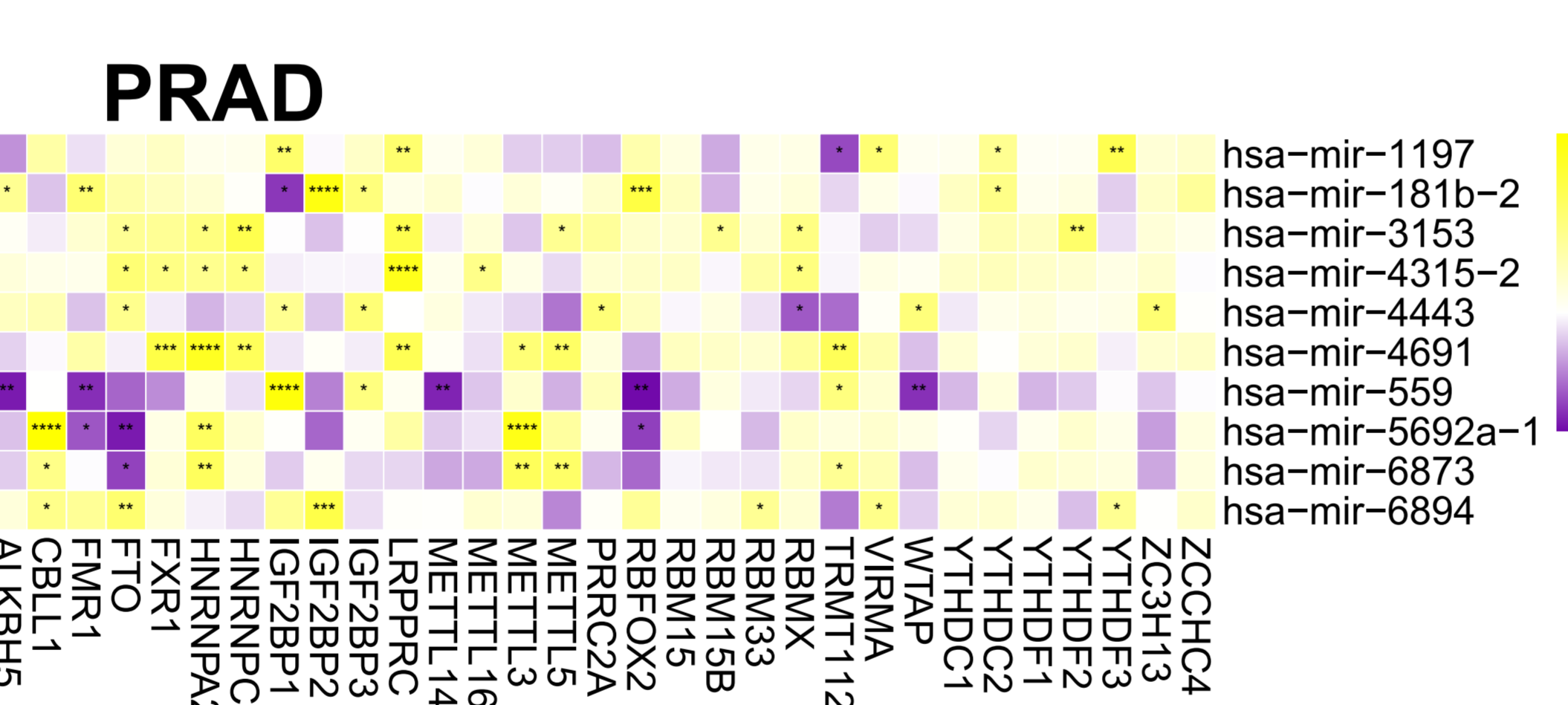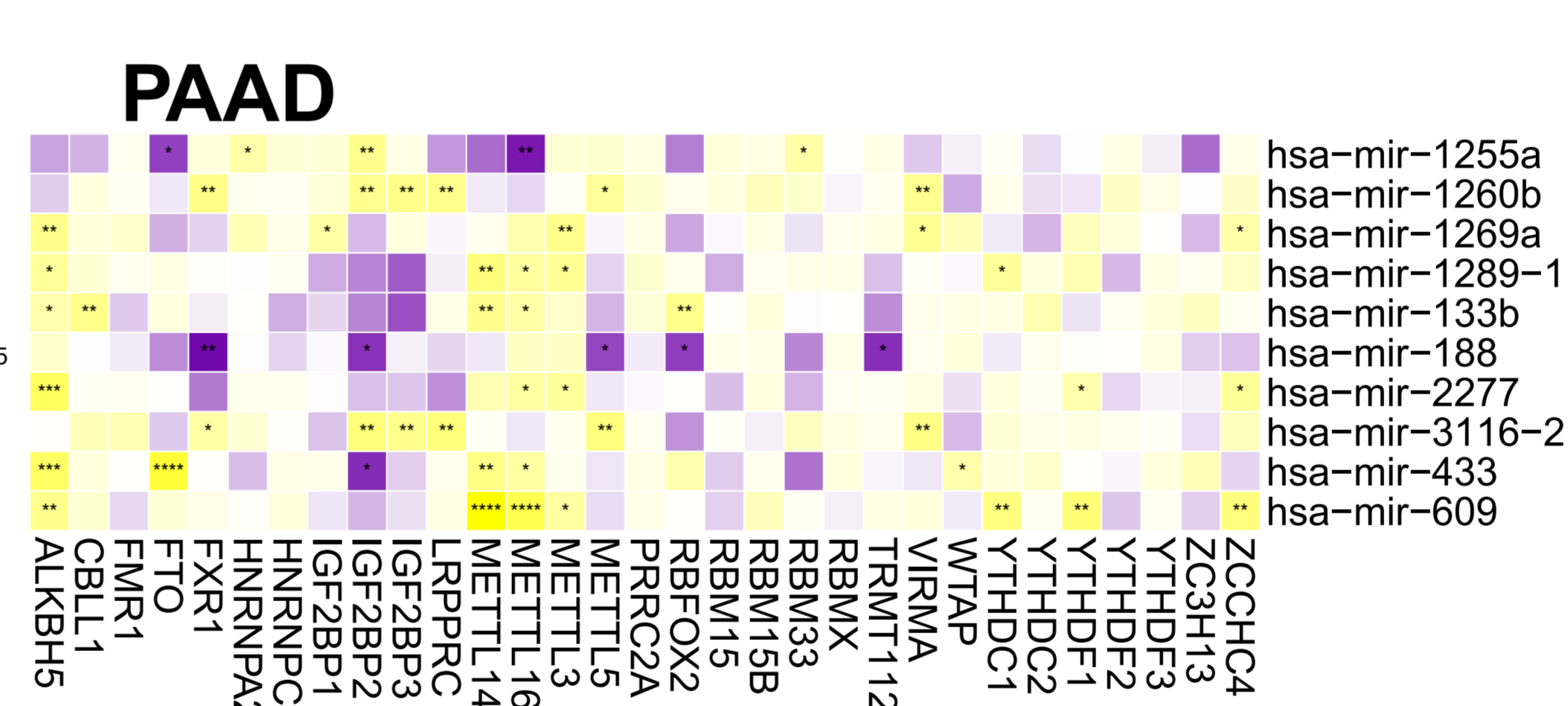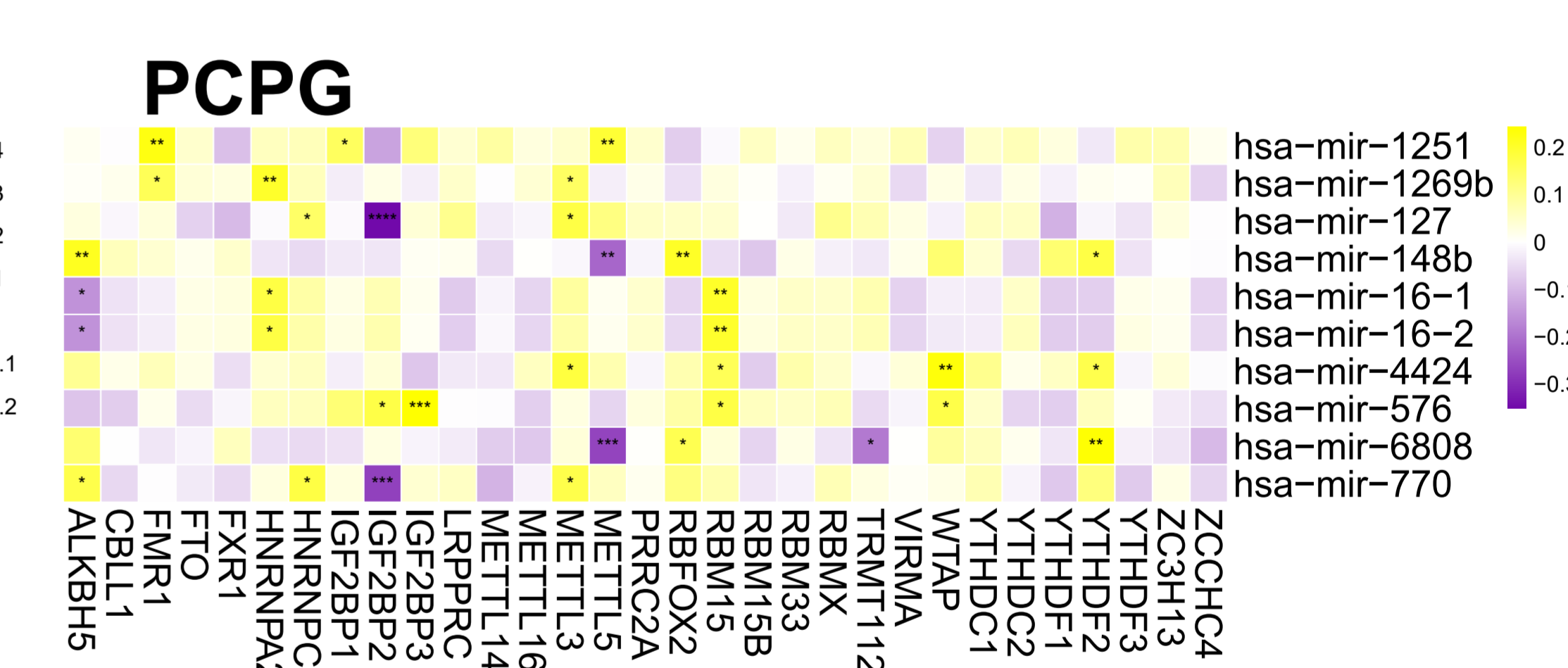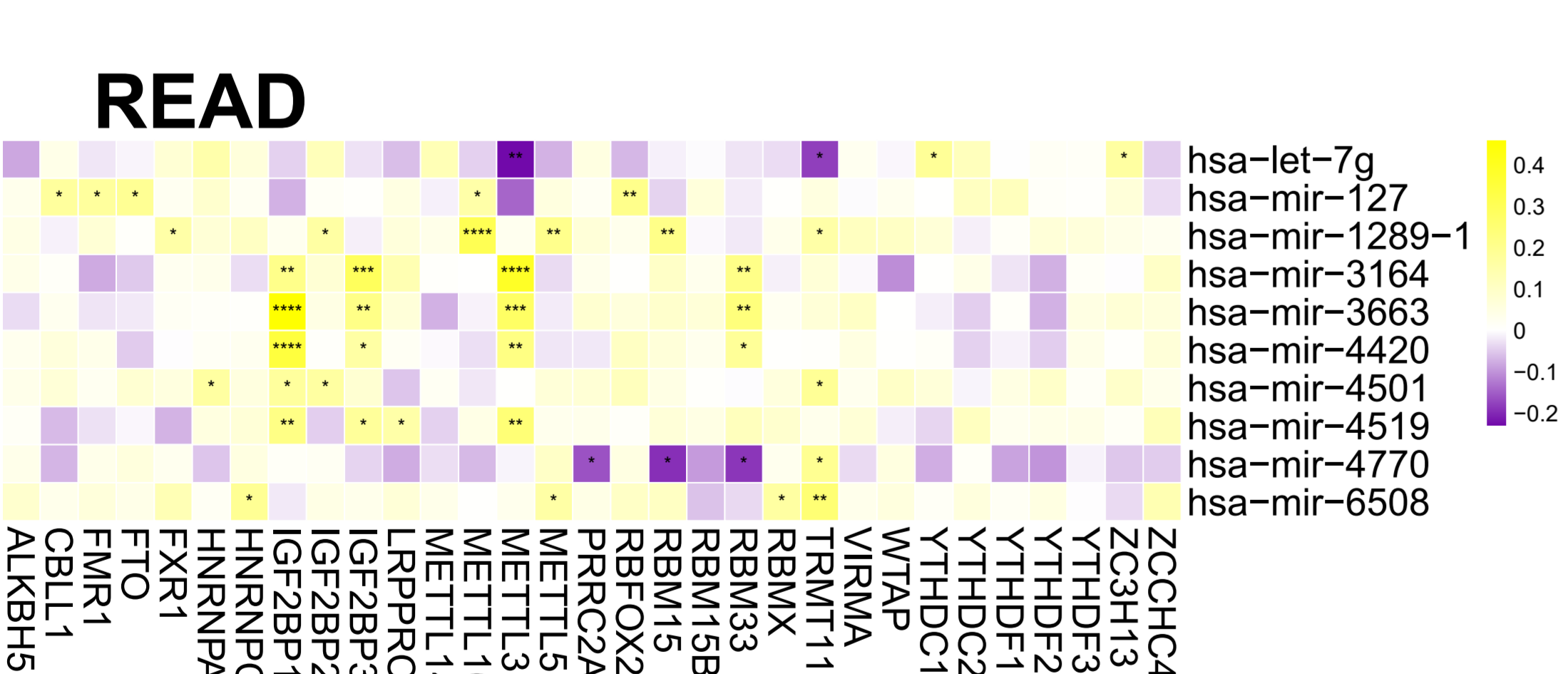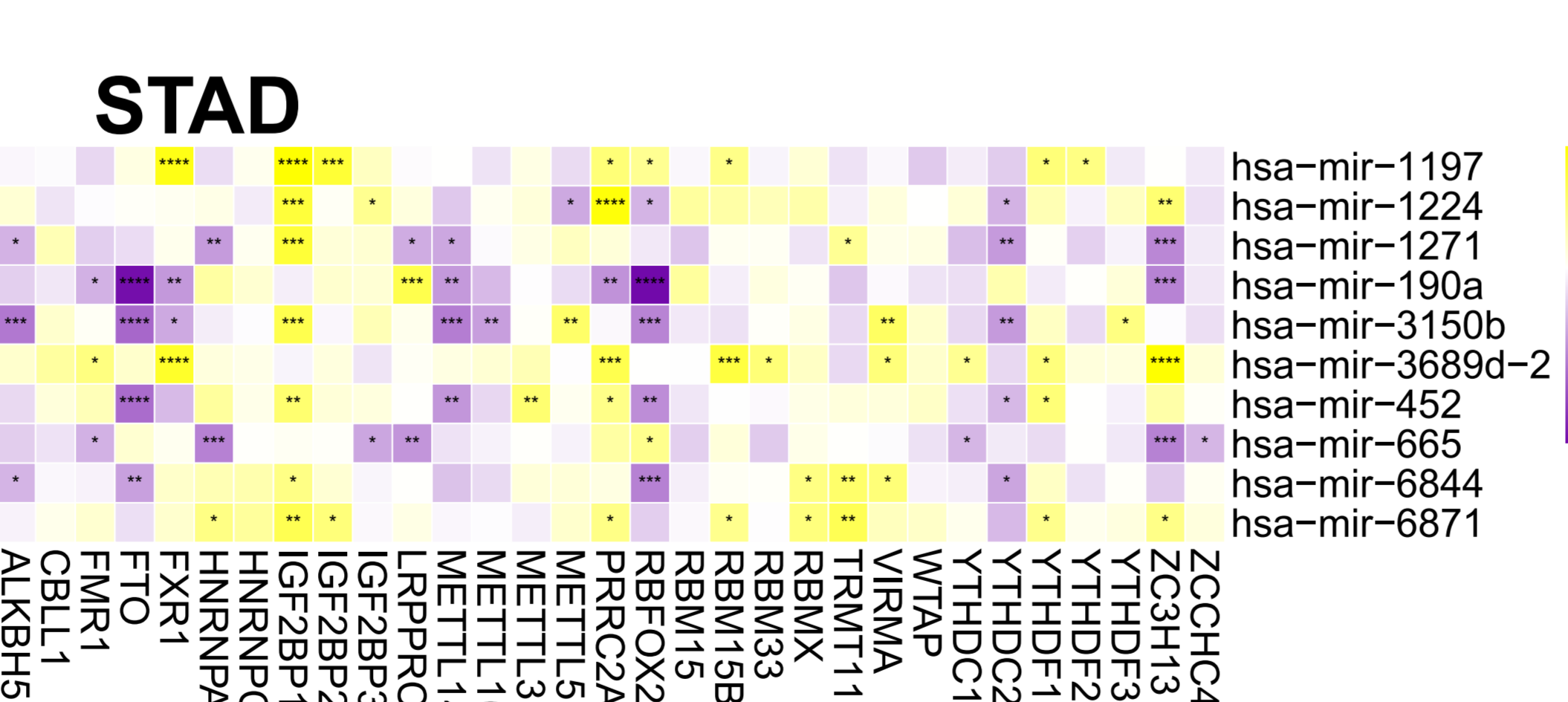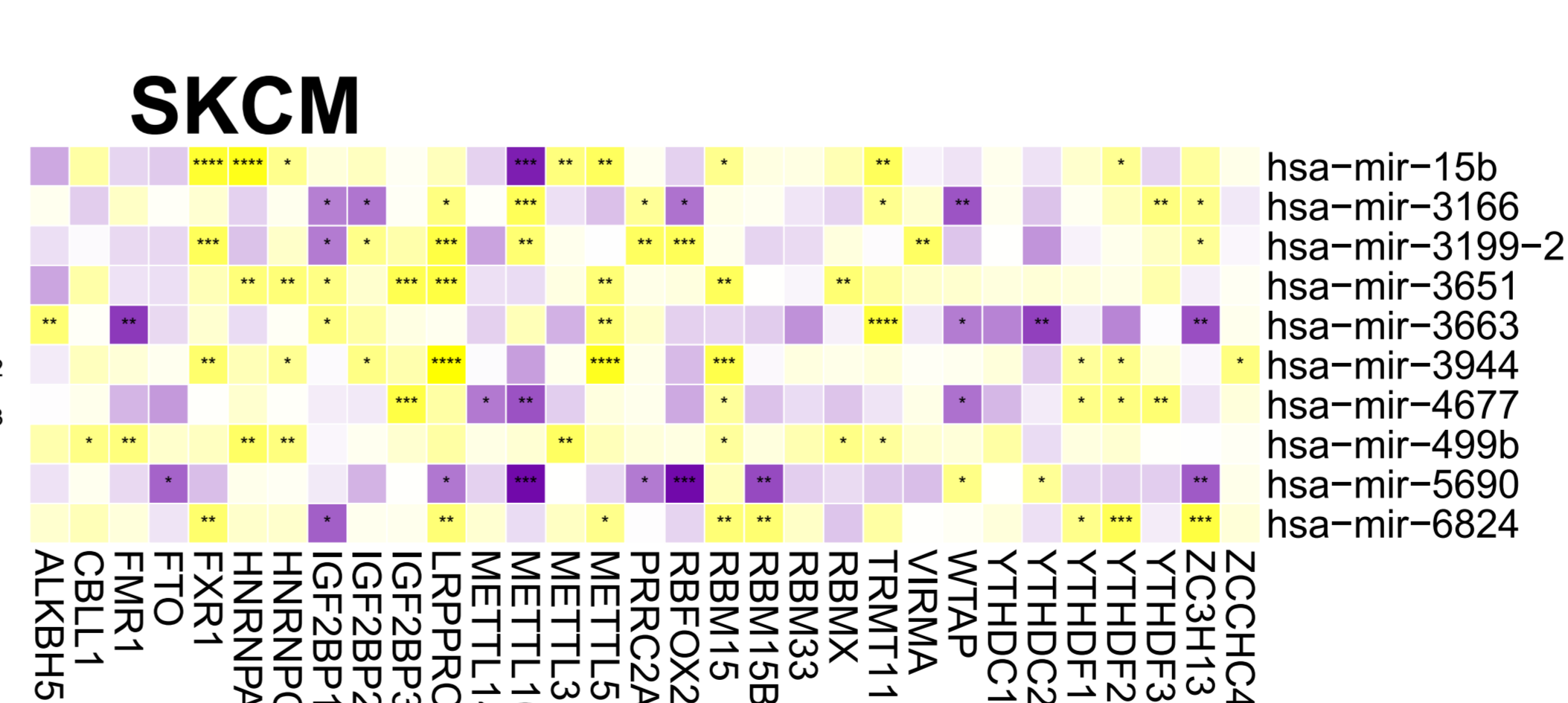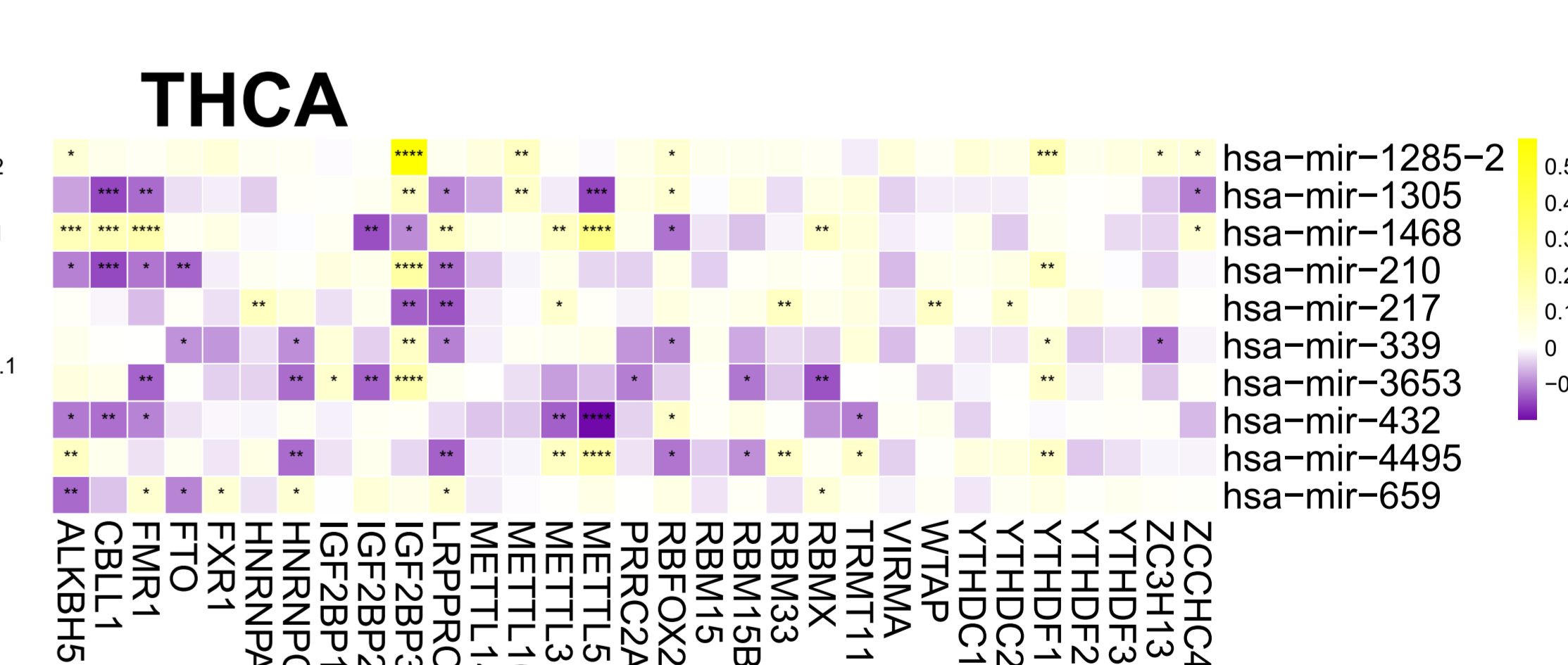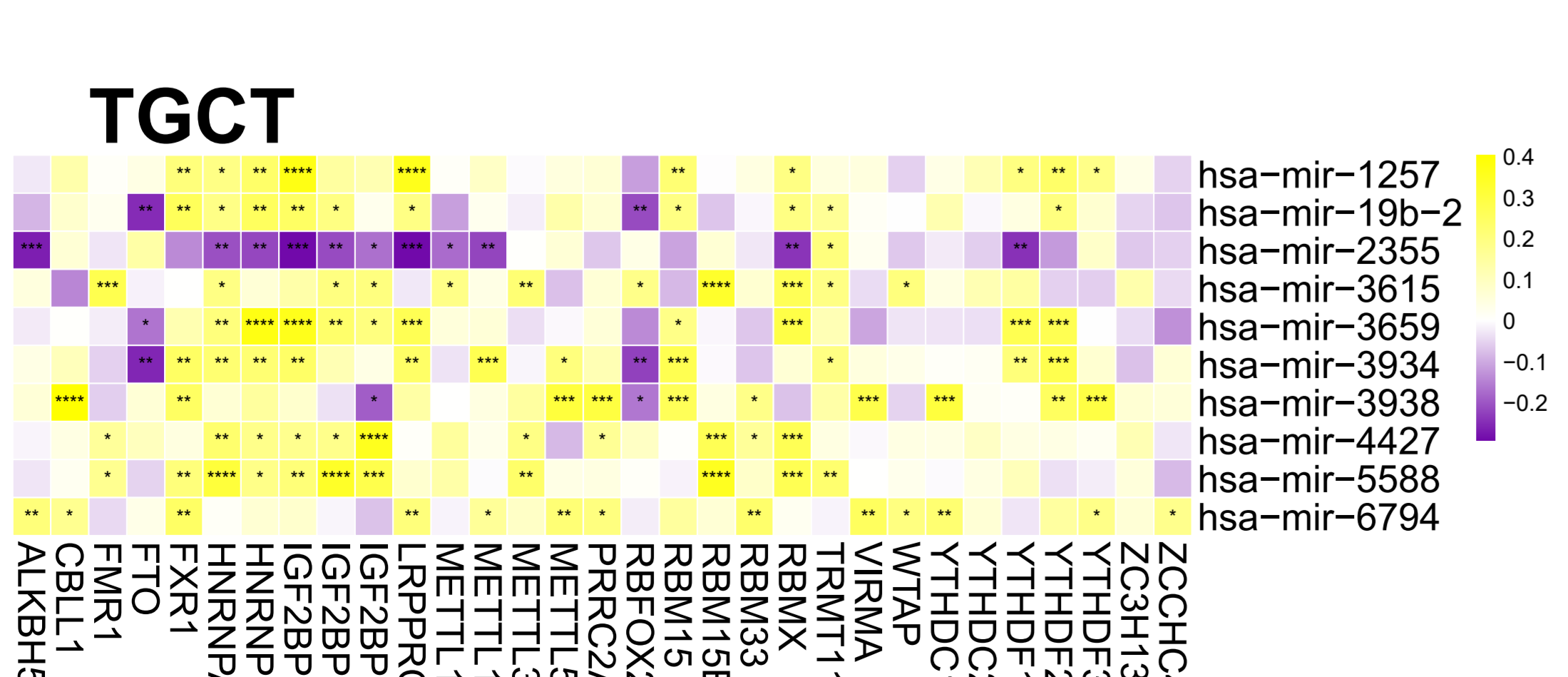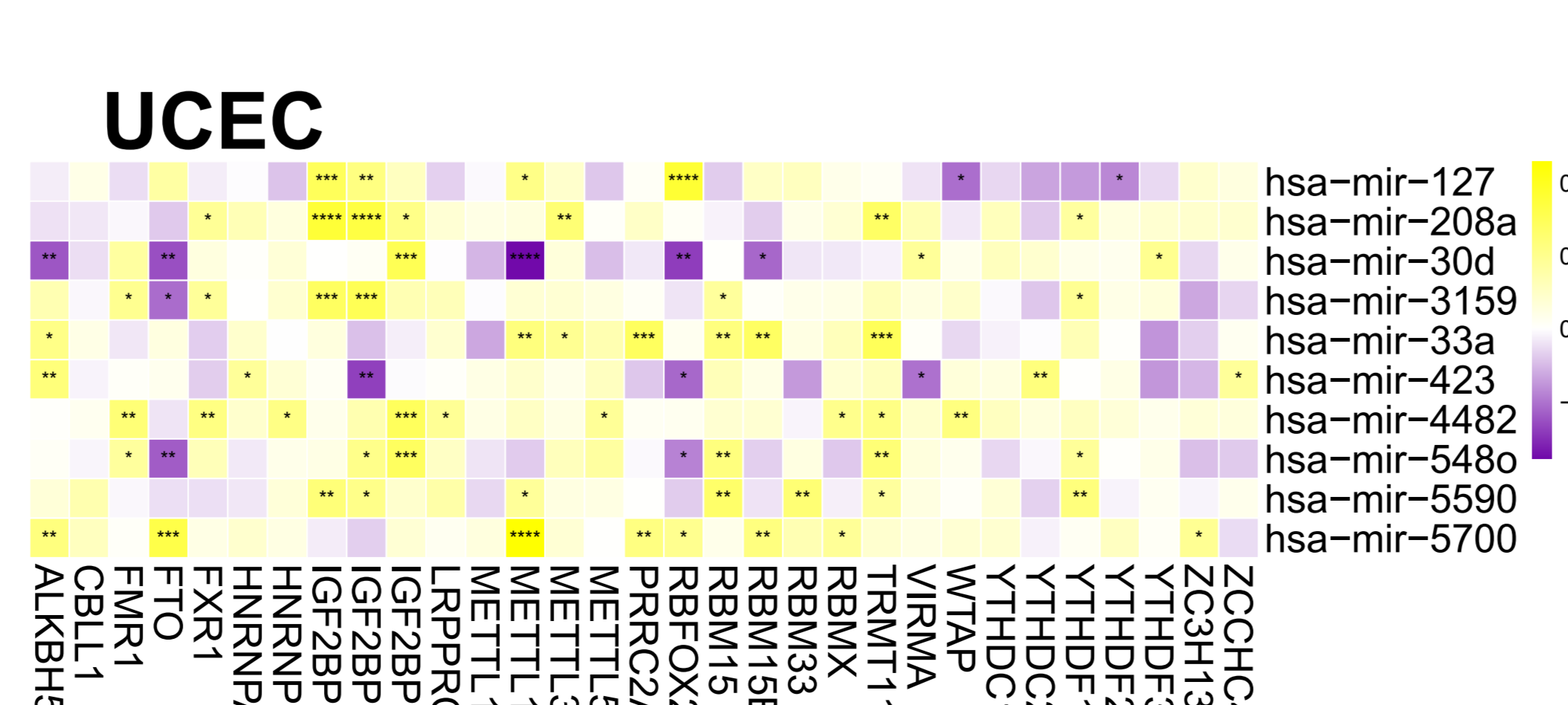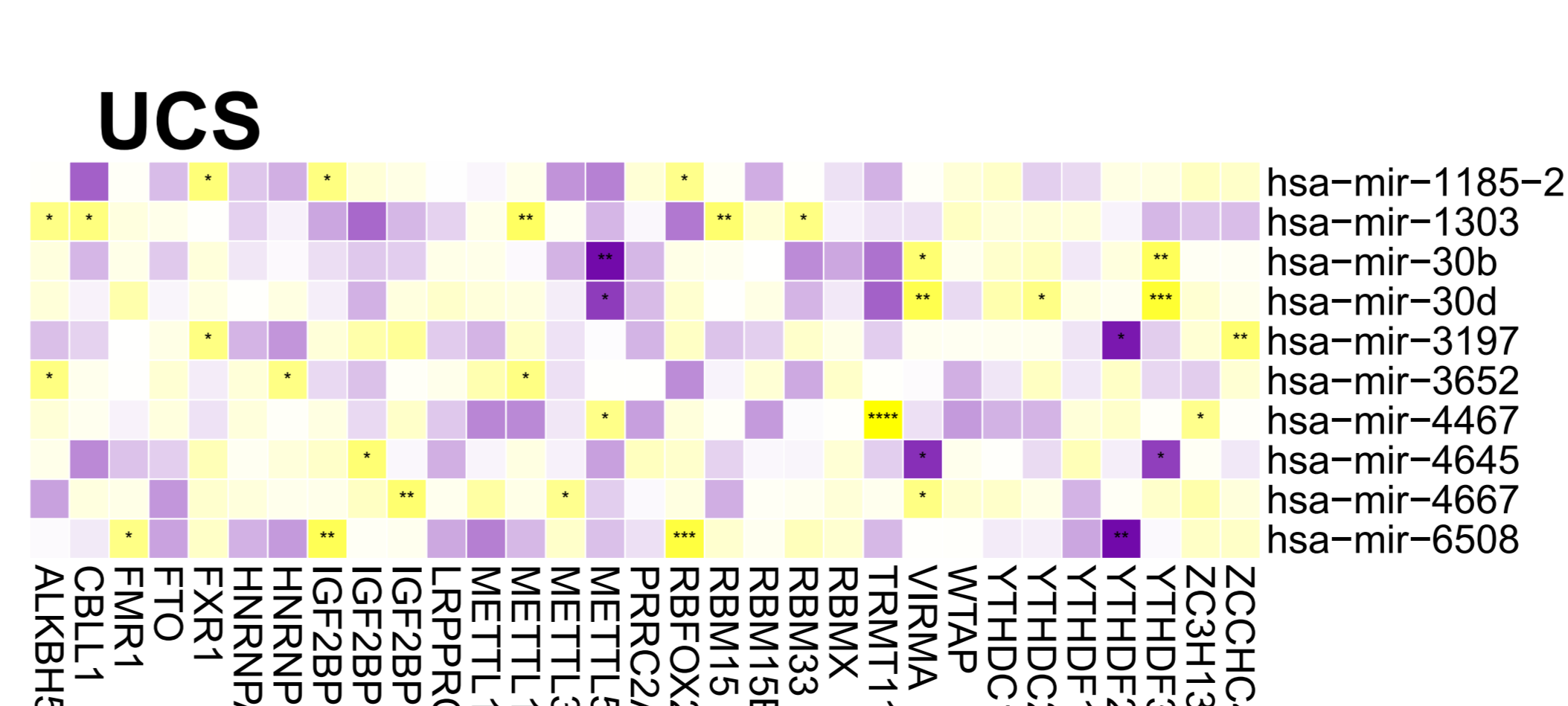

Supplement: Supplementary file 1 [file biomedicines-12-02211-s001.zip › Supplementary Files/Supplementary Figure 10.pdf]

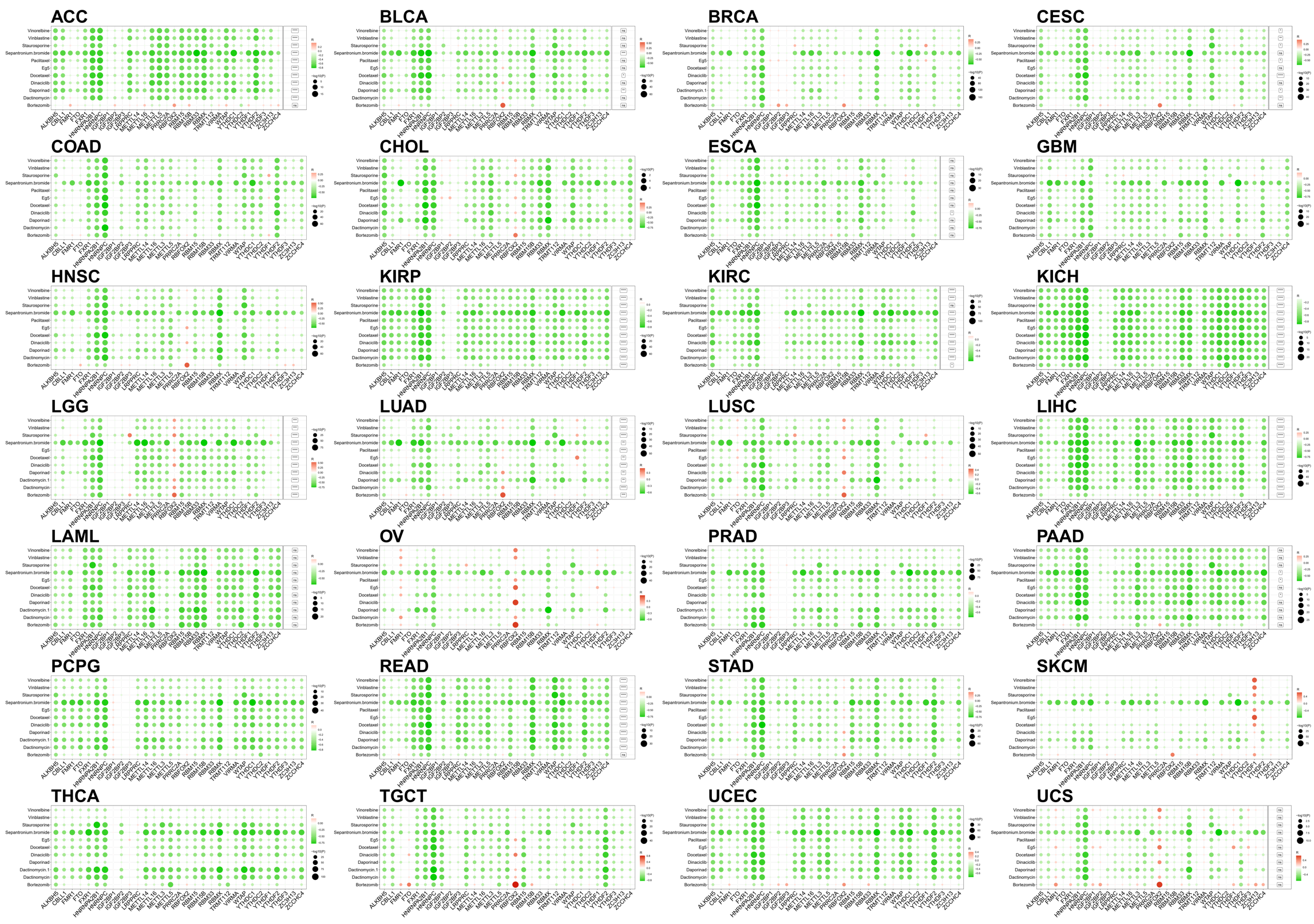

Supplement: Supplementary file 1 [file biomedicines-12-02211-s001.zip › Supplementary Files/Supplementary Figure 11.pdf]
