# Supplementary material for: Interplay of RNA m6A Modification-Related Geneset in Pan-Cancer
Source: Biomedicines. 2024 Sep 27;12(10):2211. doi: 10.3390/biomedicines12102211 (PMC11504890; doi:10.3390/biomedicines12102211)
Supplement: Supplementary file 1 [file Supplementary Figure 9.pdf › Supplementary Files/Supplementary Figure 9.pdf]

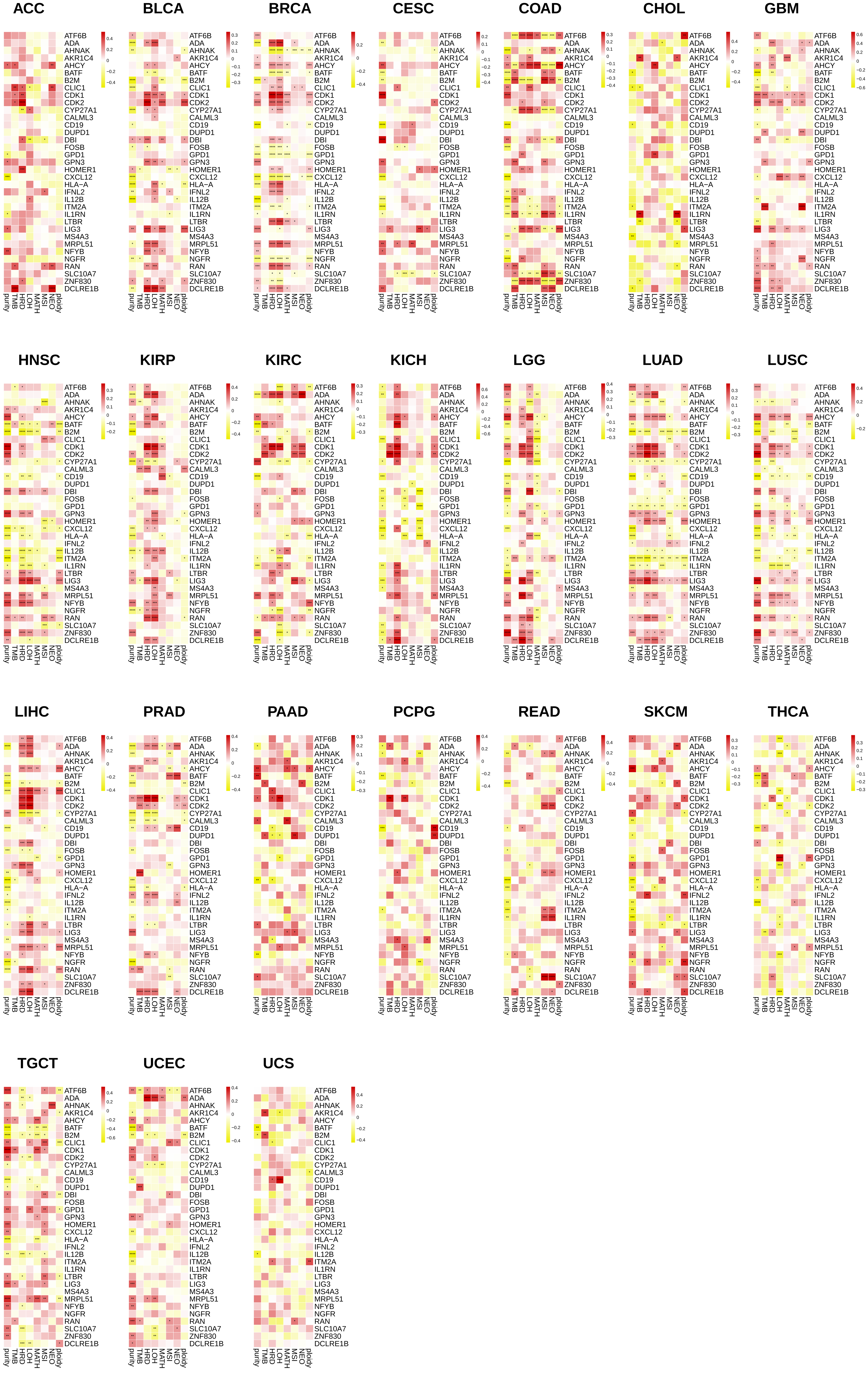
ACC
BLCA
BRCA
CESC
COAD
CHOL
GBM
HNSC
KIRP
KIRC
KICH
LGG
LUAD
LUSC
LIHC
PRAD
PAAD
PCPG
READ
SKCM
THCA
TGCT
UCEC
UCS
ATF6B
ADA
AHNAK
AKR1C4
AHCY
BATF
B2M
CLIC1
CDK1
CDK2
CYP27A1
CALML3
CD19
DUPD1
DBI
FOSB
GPD1
GPN3
HOMER1
CXCL12
HLA−A
IFNL2
IL12B
ITM2A
IL1RN
LTBR
LIG3
MS4A3
MRPL51
NFYB
NGFR
RAN
SLC10A7
ZNF830
DCLRE1B
purity
TMB
HRD
LOH
MATH
MSI
NEO
ploidy
