# Supplementary material for: Interplay of RNA m6A Modification-Related Geneset in Pan-Cancer
Source: Biomedicines. 2024 Sep 27;12(10):2211. doi: 10.3390/biomedicines12102211 (PMC11504890; doi:10.3390/biomedicines12102211)
Supplement: Supplementary file 1 [file Supplementary Figure 2.pdf › Supplementary Files/Supplementary Figure 2.pdf]

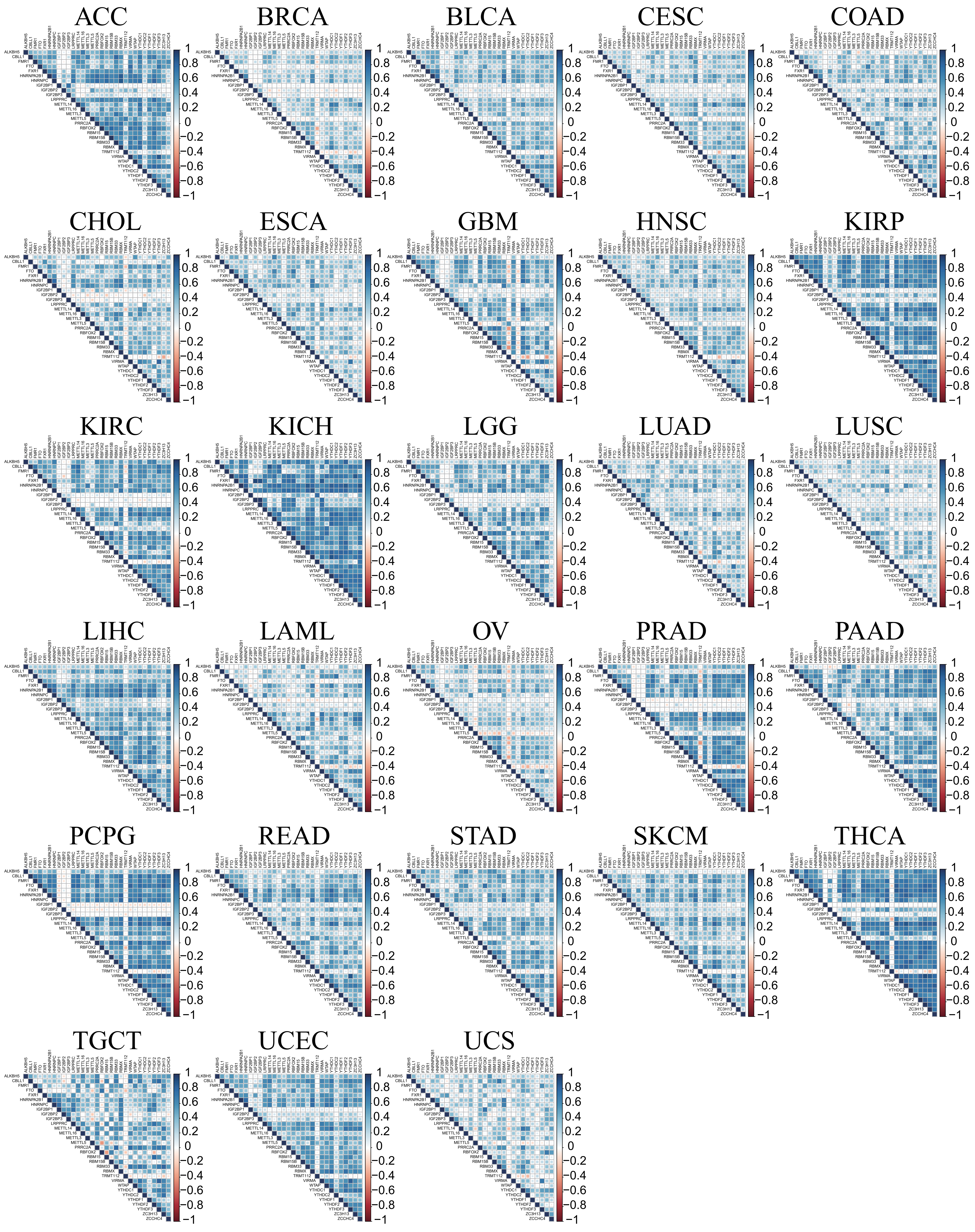
ACC
BRCA
BLCA
CESC
COAD
CHOL
ESCA
GBM
HNSC
KIRP
KIRC
KICH
LGG
LUAD
LUSC
LIHC
LAML
OV
PRAD
PAAD
PCPG
READ
STAD
SKCM
THCA
TGCT
UCEC
UCS
ALKBH5
CBLL1
FMR1
FTO
FXR1
HNRNPA2B1
HNRNPC
IGF2BP1
IGF2BP2
IGF2BP3
LRPPRC
METTL14
METTL16
METTL3
METTL5
PRRC2A
RBFOX2
RBM15
RBM15B
RBM33
RBMX
TRMT112
VIRMA
WTAP
YTHDC1
YTHDC2
YTHDF1
YTHDF2
YTHDF3
ZC3H13
ZCCHC4
1
0.8
0.6
0.4
0.2
0
−0.2
−0.4
−0.6
−0.8
−1
